# Pan-cancer analysis identifies telomerase-associated signatures and cancer subtypes

**DOI:** 10.1186/s12943-019-1035-x

**Published:** 2019-06-10

**Authors:** Zhenhua Luo, Weixu Wang, Feng Li, Zhou Songyang, Xuyang Feng, Changchang Xin, Zhiming Dai, Yuanyan Xiong

**Affiliations:** 1 Key Laboratory of Gene Engineering of the Ministry of Education and State Key Laboratory of Biocontrol, School of Life Sciences, Sun Yat-Sen University, Guangzhou, 510006 China; 2 School of Data and Computer Science, Sun Yat-Sen University, Guangzhou, 510006 China; 3 Verna and Marrs Mclean Department of Biochemistry and Molecular Biology, Baylor College of Medicine, One Baylor Plaza, Houston, TX 77030 USA; 4 Division of Gastroenterology, Hepatology and Nutrition, Cincinnati Children’s Hospital Medical Center and the Department of Pediatrics Cincinnati, Ohio, 45229 USA; 5 Department of Cancer Biology, University of Cincinnati, Cincinnati, OH 45230 USA; 6 College of Forestry and Landscape Architecture, South China Agricultural University, Guangzhou, China; 7 The First Affiliated Hospital, Sun Yat-sen University, Guangzhou, 510275 China

**Keywords:** Telomerase, Signature, Survival, Drug target

## Abstract

**Background:**

Cancer cells become immortalized through telomere maintenance mechanisms, such as telomerase reverse transcriptase (TERT) activation. In addition to maintaining telomere length, TERT activates manifold cell survival signaling pathways. However, telomerase-associated gene signatures in cancer remain elusive.

**Methods:**

We performed a systematic analysis of TERT high (TERT^high^) and low (TERT^low^) cancers using multidimensional data from The Cancer Genome Atlas (TCGA). Multidimensional data were analyzed by propensity score matching weight algorithm. Coexpression networks were constructed by weight gene coexpression network analysis (WGCNA). Random forest classifiers were generated to identify cancer subtypes.

**Results:**

The TERT^high^-specific mRNA expression signature is associated with cell cycle-related coexpression modules across cancer types. Experimental screening of hub genes in the cell cycle module suggested TPX2 and EXO1 as potential regulators of telomerase activity and cell survival. MiRNA analysis revealed that the TERT^high^-specific miR-17-92 cluster can target biological processes enriched in TERT^low^ cancer and that its expression is negatively correlated with the tumor/normal telomere length ratio. Intriguingly, TERT^high^ cancers tend to have mutations in extracellular matrix organization genes and amplify MAPK signaling. By mining the clinical actionable gene database, we uncovered a number of TERT^high^-specific somatic mutations, amplifications and high expression genes containing therapeutic targets. Finally, a random forest classifier integrating telomerase-associated multi-omics signatures identifies two cancer subtypes showed profound differences in telomerase activity and patient survival.

**Conclusions:**

In summary, our results depict a telomerase-associated molecular landscape in cancers and provide therapeutic opportunities for cancer treatment.

**Electronic supplementary material:**

The online version of this article (10.1186/s12943-019-1035-x) contains supplementary material, which is available to authorized users.

## Background

Telomeres are the terminal ends of linear chromosomes, and in most eukaryotes, they are composed of a GT-rich DNA repeat sequence (TTAGGG) and capped by telomere-specific binding proteins [1]. Telomeres have an essential role in the protection of chromosome ends and prevent them from being recognized as DNA damage sites [2]. Telomeres shorten progressively with each cell proliferation, causing cell senescence and eventual death [3–5]. Maintenance of telomere length enables cells to bypass crises and become immortalized [6].

Most cancers activate telomerase to maintain telomere length [7]. Telomerase consists of two main components, enzymatic subunit telomerase reverse transcriptase (TERT) and RNA subunit TERC. Telomerase binds to the telomere G strand and counteracts telomere shortening by adding telomere repeats during unlimited proliferation [7]. In addition, growing evidence reveals multiple noncanonical functions of TERT. For example, TERT activates NF-κb and WNT signaling by functioning as a transcription cofactor [8–10]. Interaction between TERT and NF-κb subunit p65 modulates TERT nuclear translocation in myeloma cells [11]. Moreover, TERT increases cell adhesion and migration independent of telomerase activity [12]. All these canonical and noncanonical functions provide cell survival signals and promote tumorigenesis. Therefore, TERT plays a pivotal role in cancer by bridging various regulatory machinery.

Although TERT is actively transcribed in most human normal cells and tissues, TERT transcripts in normal cells are non-functional variants generated by alternative splicing [13]. However, cancer cells are able to produce full length of functional TERT and develop numerous regulatory mechanisms of TERT activation and stabilization. These mechanisms include TERT promoter point mutations, methylation, rearrangements, DNA amplifications, transcript fusions and posttranslational modification [14–19]. In addition to telomerase activation, there is an alternative telomere maintenance mechanism involving a homologous recombination–based process, termed alternative lengthening of telomeres (ALT), which is found in 5% of tumors [20, 21].

A recent systemic analysis of 18,430 samples across 31 cancer types has discovered that approximately 22% of tumors without detectable TERT expression might be absent from the two mechanisms of telomere maintenance [18]. This type of tumor can serve as an excellent control for the investigation of gene signatures linked to TERT expression or telomerase activation. Although previous studies have revealed that telomerase activity is able to be estimated by the expression of a group of genes [18], a study integrating genome-wide alterations with TERT expression would provide deep insights into the mechanisms associated with telomerase activity and its related signaling circuits.

To address these questions above, we assembled genome-wide molecular data across 8 cancer types in The Cancer Genome Atlas (TCGA) and performed comprehensive pan-cancer analysis by comparing TERT high (TERT^high^) and low (TERT^low^) groups. We identified a common cell cycle pathway associated with TERT expression and experimentally validated that knockdown of TPX2 or EXO1 diminished telomerase activity. Examination of the telomerase gene signatures uncovered multiple clinically actionable genes. In addition, we show that these telomerase gene signatures are predictive of survival.

## Methods

### Propensity score matching weight algorithm

We collected TCGA multi-omics data and clinical characteristics (sex, race, alcohol, laterality, purity and so on) for 8 cancers from the cBioPortal and then applied a propensity score matching (PSM) analysis to identify significant molecular features. Propensity score matching is widely used for studying treatment efforts in observational studies. We used the propensity score method proposed by Liang Li [22]. Unlike pair matching, this method improves balance and estimates efficiency and uses all subjects by weighting them so that every subject potentially contributes to the estimation.

We first calculated the propensity score using logistic regression with “TERT^high^/TERT^low^ status” as the responsible variable. Through the propensity scoring method, we can balance the covariable and use standardized difference to examine the balance (< 10%, Additional file 1: Figure S1). Among all variables, the race variable in KIRP is > 10% after the propensity scoring model was applied. Using the chi-square test, we found that there was no significant difference in this variable between the two groups (*P* = 0.43).

After completing the above procedure, we compared the molecular data between the two balanced groups (TERT^high^ and TERT^low^) by supplying the sample weights calculated from propensity scoring analysis to a linear regression model using TERT^high^/TERT^low^ status as the sole independent variable. Furthermore, we calculated the corresponding FDR adjusted *P* value of the TERT^high^/TERT^low^ status effect. To measure the TERT^high^/TERT^low^ effects and reduce the bias of confounders, we used a matching weight (MW) estimator instead of the gene’s fold change. The MW estimator formula was as follows:

$$ {\triangle}_{\mathrm{MW}}=\frac{\sum \limits_{i=1}^n{X}_i{Z}_i{Y}_i}{\sum \limits_{i=1}^n{X}_i{Z}_i}-\frac{\sum \limits_{i=1}^n{W}_i\left(1-{Z}_i\right){Y}_i}{\sum \limits_{i=1}^n{W}_i\left(1-{Z}_i\right)} $$

where *Y*_*i*_ *Z*_*i*_ *W*_*i*_ denotes the outcome (mRNA expression level; SCNA; methylation data; miRNA expression level; SNP mutations), 1 o 0 denotes the TERT^low^ group or TERT^high^ group, and the mean matching weight was assigned to the ith subject. To examine the robustness of the signal, we performed resampling by randomly selecting > 30 samples in both groups (TERT^high^ and TERT^low^). Applying the PSM algorithm to this resampled group, we found that the MW estimators for all mRNAs were highly correlated with those of the original sample set (Pearson correlation R = 0.83–0.97, *P* < 2.2e-16). To ensure that our significant feature sets were not caused by random noise, we also computed the *P* value by a random permutation test (randomizing the TERT^high^/TERT^low^ status of all samples from the same individual each time). We only retained the significant features (*P* value < 0.05) for further analysis (Additional file 2: Figure S2). For different molecular signatures, TERT^high^/TERT^low^ specificity was determined by the relative levels of molecular signatures.

### mRNA and miRNA expression analysis

We obtained normalized mRNA expression data in RSEM (RNA-seq by expectation maximization from cBioPortal. After log2 transformation, we selected mRNAs with an average expression level > 1 in both the TERT^high^ and TERT^low^ groups. Applying the propensity score algorithm and permutation test, we identified significantly expressed mRNAs in the TERT^high^ and TERT^low^ groups (FDR < 0.05, permutation test *P* value < 0.05).

We obtained miRNA expression data (in read per million) from Firehose. The propensity score algorithm and permutation test were used to identify the miRNAs that showed significant differences between the TERT^high^ and TERT^low^ groups (FDR < 0.05, permutation test *P* value < 0.05).

We curated miRNA targets from the following well-established databases: miRtarBase, miRanda and miRDB. We extracted the intersect of targets in miRanda and miRDB and merged these genes with the targets in miRtarBase as our final miRNA targets. We then selected the strongest targets that are hit by at least two miRNAs in each group (≥2 for upregulated miRNAs or miRNAs, ≥4 for downregulated miRNAs due to the larger number of miRNAs in this group).

### DNA methylation analysis

We obtained DNA methylation HM450 data from the cBioPortal. For genes with multiple methylation probes, we selected methylation probes showing the strongest anti-correlation with mRNA gene expression data. Then, we applied the PSM model to find significant TERT^high^/TERT^low^ -specific methylation probes at an FDR < 0.05 and a permutation test *P* < 0.05. In addition, to determine the potential regulatory relationship between TERT^high^/TERT^low^ -specific methylation probes and TERT^high^/TERT^low^ -specific expressed genes, we used Fisher’s exact test to assess whether TERT^high^/TERT^low^ -specific methylation probes are significantly enriched in TERT^high^/TERT^low^ -specific expressed genes.

### Weighted gene co-expression network analysis (WGCNA) for mRNA and miRNA

We constructed the mRNA co-expression network using ‘WGCNA’ R package with entire datasets [23]. First, the pearson correlations were calculated between each pair of genes to obtain similarity matrix. Then, WGCNA used power function to transform the similarity matrix to adjacency matrix. The key parameter, beta, for scale-free weighted network construction, was determined by the scale-free topological fit test. We chose a scale free fit R^2 > 0.9 to obtain a high-confidence scale free network. Further, the pair-wise topological overlap (TO) between genes was calculated to obtain co-expression modules using cutreeDynamic function in the dynamicTreeCut R package. The expression of each module was summarized by module eigengene (ME). Highly correlated modules were further merged by mergeCloseModules function in WGCNA R package. The gene’s connectivity was defined by the sum of edge weights and calculated by intramodular Connectivity function in WGCNA R package. The hub genes of each module are defined as the top 5% of the genes with the highest connectivity in each module. For each module, we defined the module membership measure (kME), which is module eigengene based connectivity, as the correlation between gene expression values and module eigengene.

We utilized the module preservation statistic Zsummary, described in the ‘modulePreservation’ R function implemented in WGCNA, to assess the overlap between network modules obtained from the whole samples and TERT^high^ or TERT^low^ samples. The Zsummary statistic takes into account the overlap in module membership, the density (mean connectivity) and connectivity (sum of connections) patterns of modules. A module is considered not being preserved if preservation Zsummary< 2, moderately preserved if 2 ≤ Zsummary< 10, and highly preserved if Zsummary ≥10.

### Somatic mutation and somatic copy number variation data analysis

We obtained the mutation data (MAF file) and significant SCNA (both focal- and arm-level) from Firehose. For mutation data, we only retained samples with < 1000 mutations in their exomes for further analysis. We focused on the mutations with ≥5% mutation frequency in patients. After combining the high confidence SNP and SCNA, we applied propensity score model to identify the TERT^high^/TERT^low^ -specific SNP and SCNA at FDR < 0.05 and permutation test *P* < 0.05.

### Gene ontology and pathway analysis

Gene ontology analysis was performed using EnrichGO function in clusterProfiler R package [24], with the following parameters:ont = “MF”, pvalueCutoff = 0.01, qvalueCutoff = 0.05. False-discovery rate adjusted *P* values were calculated using Benjamini-Hochberg correction. Pathway analysis was performed using Toppgene with TERT^high^-specific mutated or amplified genes as input [25].

### Other data preparation

Tumor/normal telomere length ratios of cancer samples were downloaded from Barthel et al. [18]. Gene list for telomerase activity estimation was obtained from Barthel [18]. FDA-approved drugs and their targets information were downloaded from Drugbank database [26].

### Random forest clustering algorithm for tumor subtype identification

We used two different sets of molecular signatures as molecular markers for unsupervised classification: 1. “global markers”, which consist of consensus hub genes of the cell cycle/mitosis nuclear division module, and miRNAs belong to the miR-17-92 cluster. 2. “cancer-specific markers”, which contains TERT^high^/TERT^low^ -specific top20 expressed genes (including TERT) and DNA methylation probes, and the top 5 expressed miRNAs, SNPs and SCNAs. We combined the above molecular markers and generated a molecular marker profile for each cancer.

We applied a random forest clustering algorithm proposed by Tao Shi and Steve Horvath [27] that uses a random forest algorithm to perform a dissimilarity measure for 11n labeled data. This algorithm handles mixed variable types well and is invariant to monotonix transformations of the input variable. First, we obtained a similarity matrix from an ensemble of individual tree predictors (terminal node) that distinguish observed from ‘synthetic’ data. The observed data are original data, while the synthetic data are collected by randomly sampling from the product of the empirical marginal distribution of the variable. A synthetic class outcome is defined by labeling the observed data as class 1 and the synthetic data as class 2. Then, at the random forest classification step, one can define a similarity measure between unlabeled observations. For each tree, if observations i and j both land in the same terminal node, the similarity between i and j is increased by one. At the end of the forest construction, the similarities are symmetrized and divided by the number of trees. Then, we calculated dissimilarity between i and j, which is defined as RF dissimilarityij = sqrt(1 - RF similarityij). We used the RF dissimilarity as input for partitioning around medoids (PAM) clustering, which is implemented in the R function Pam in the package cluster. Finally, we obtained new clusters (or subtypes) for each cancer type. The random forest algorithm was implemented with the R package ‘randomForest’.

### Cell lines

Human embryonic kidney HEK293T, human liver hepatocellular carcinoma HepG2 cells and human fibrosarcoma HTC75 cells were maintained in high-glucose DMEM (Hyclone, SH30243) with 10% fetal bovine serum (Hyclone, SH30070) and 1% penicillin/streptomycin. Mycoplasma testing was performed by PCR every month to ensure non-contamination. The general length of time between thawing and use in the described experiments is 1 week.

### RNAi experiments and quantitative RT-PCR

We performed RNAi experiments and quantitative RT-PCR as described previously [28–30]. Cells were transfected with appropriate siRNA oligos (from Ribobio) using RNAiMax (Invitrogen) according to the manufacturer’s instructions. Cells were harvested and examined 48 h after transfection. Real-time quantitive RT-PCR was carried out to confirm inhibition of mRNA expression. Briefly, total RNA was isolated with the TRIzol reagent (Invitrogene), reverse transcribed using the RevertAid First Strand cDNA Synthesis Kit (Thermo Fisher), and then amplified using the ABI StepOnePlus real-time PCR system (Applied Biosystems). Cycling conditions were 40 cycles of 95 °C for 15 s and 60 °C for 60 s.

Sequences of the various siRNAs used in the study were:

siTPX2: 5′-AUGAAAGUUUCUAACAACAAATT-3′

siEXO1: 5′- CAAGCCUAUUCUCGUAUUUTT-3′

siFOXM1: 5′- GCCAAUCGUUCUCUGACAGAATT-3′

si-RAD54L: 5′- UGUAAUUCGACACCAGCACTT-3′

si-NEIL3: 5′- UAUCCGAUGAAAUACAUAGTT-3′

Sequences of the various Q-PCR primers used in the study were:

- TPX2-Q-FP: 5′- ATGGAACTGGAGGGCTTTTTC-3′ TPX2-Q-RP: 5′- TGTTGTCAACTGGTTTCAAAGGT-3′
- EXO1-Q-FP: 5′- CCTCGTGGCTCCCTATGAAG-3′ EXO1-Q-RP: 5′- AGGAGATCCGAGTCCTCTGTAA-3′
- FOXM1-Q-FP: 5′- GGAGCAGCGACAGGTTAAGG-3′ FOXM1-Q-RP: 5′- GTTGATGGCGAATTGTATCATGG-3′
- RAD54L-Q-FP: 5′- ATGGAACTGGAGGGCTTTTTC-3′ RAD54L-Q-RP: 5′- TGTTGTCAACTGGTTTCAAAGGT-3′
- NEIL3-Q-FP: 5′- CAAGCGTCCTAATTGTGGTCA-3′ NEIL3-Q-RP: 5′- CCCTGCTAGATGTCCAACTGATT-3′

### Real-time quantitative PCR-based TRAP (Q-TRAP)

Real-time quantitative PCR-based TRAP assays were carried out as previous described [29]. Briefly, cells (3–10× 106) were lysed in 5× pellet volume of high-salt buffer (20 mM Hepes at pH 7.9, 0.42 mM KCl, 25% (vol/vol) glycerol, 0.2% Nonidet P-40, 0.1 mM EDTA, 1 mM DTT, and protease inhibitors), and then diluted with 5× volume of low-salt buffer (20 mM Hepes at pH 7.9, 100 mM KCl, 25% (vol/vol) glycerol, 0.1 mM EDTA, 1 mM DTT, and protease inhibitors) and centrifuged at > 14,000×g for 10 min at 4 °C. The supernatant was then diluted two- to five fold before being used for real-time quantitative PCR-based TRAP assay. Each 20 μL of real-time quantitative PCR-based TRAP reaction contained 1 μL of the eluted proteins, 100 ng each of TS primer (5′-AATCCGTCGAGCAGAGTT-3′) and ACX primer (5′-GCGCGGCTTACCCTTACCCTTACCCTAACC-3′), and 1 mM EGTA in SYBR Green PCR Master Mix (Applied Biosystems). The reaction mixtures were incubated at 30 °C for 30 min and then PCR amplified (40 cycles of 95 °C for 15 s and 60 °C for 60 s) by using an ABI StepOnePlus Real-Time PCR System (Applied Biosystems).

## Results

### Overview of molecular signature differences between TERT high (TERT^high^) and low (TERT^low^) cancers

To identify telomerase-associated molecular signatures, we first defined two cancer types based on TERT expression: 1) patients with high expression of TERT (TERT^high^) and 2) patients with low expression of TERT (TERT^low^). We excluded ALT cancers because of their small sample size (~ 5% of all samples in TCGA) and unique genomic and transcriptomic features that may confound the analysis. Samples with 2 or more RNA-Seq quantified reads of TERT were defined as TERT^high^, while those with less than 2 reads of TERT and without known ALT-related somatic alterations (ATRX or DAXX mutations, deletions or structural variants) were defined as TERT^low^.

**Fig. 1:**
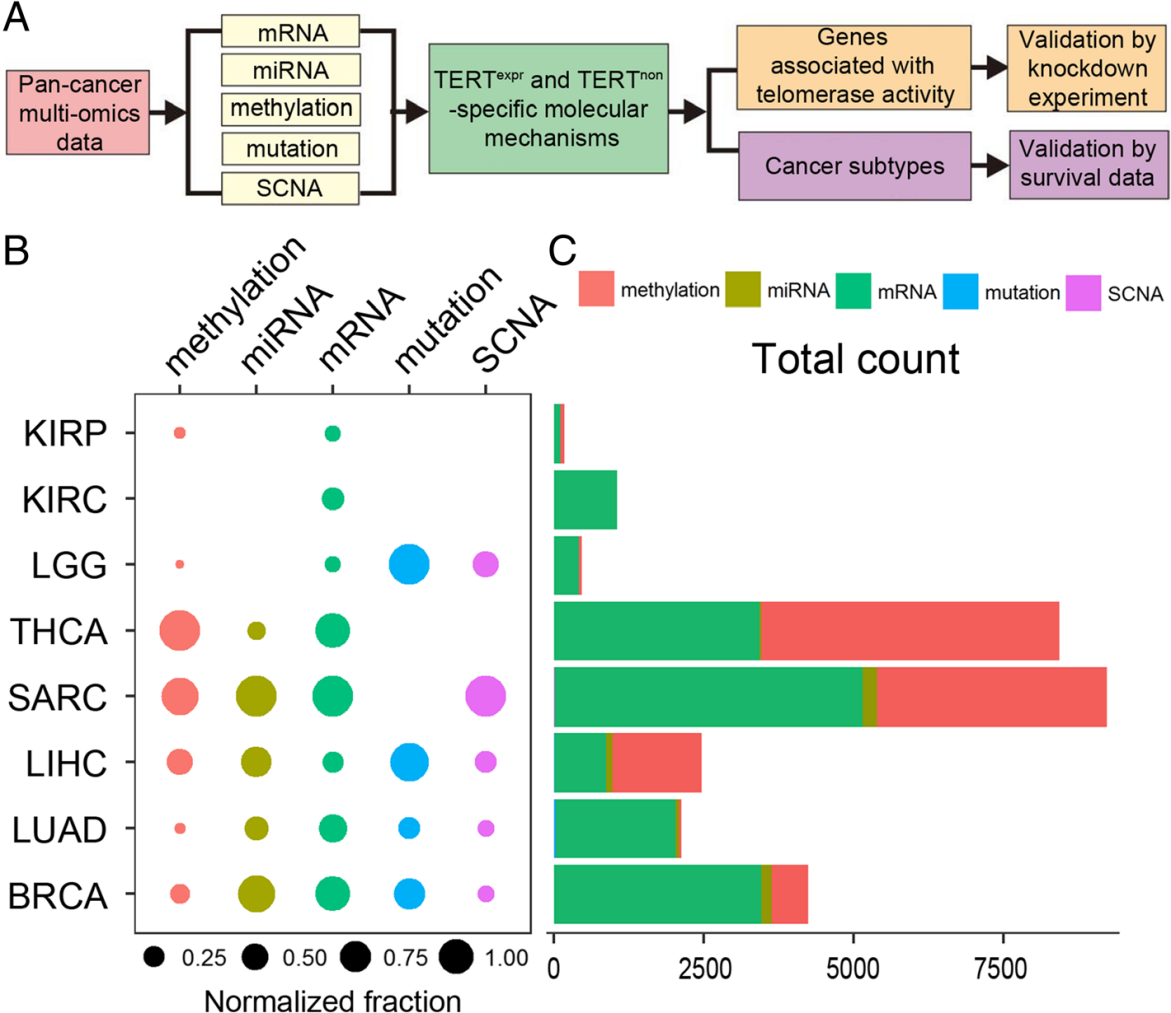
TERT^high^/TERT^low^ -specific molecular patterns across cancer types. **a** Analytic pipeline of this study. **b** Relative abundance of molecular signatures identified by the propensity scoring algorithm across cancer types (FDR < 0.05) in comparison of TERT^high^ and TERT^low^ groups. The fraction of significant features over total features was first calculated in each cancer type and then normalized across all cancer types. The bar plot shows the number of significant features for each cancer type. **c** Pie charts show the relative proportion of significant features over total features for each cancer type in comparisons of TERT^high^ and TERT^low^ groups

To investigate telomerase-associated molecular signatures across cancers, we developed an analytic pipeline by integrating multi-omics data and experimental studies (Fig. 1a). We adopted propensity score modeling (PSM) to identify molecular differences between TERT^high^ and TERT^low^ patients with removal of other confounder effects that may bias findings (e.g., age, race, gender, vital status, tissue source site, grade, laterality, BMI, histology, purity, alcohol history, see potential confounders surveyed in Additional file 1: Figure S1). Propensity score modeling is a widely used statistical technique for estimation of treatment effects and reduction in bias caused by covariables [22]. It corrects the confounder effect by balancing the propensity score. It has been shown to outperform other methods, including the t-test, ANOVA and GLM [31]. Due to the requirement of sample size (at least 30 samples in each group) for the PSM algorithm, we focused on 8 TCGA cancer types with sufficient sample size for 5 molecular types, including somatic mutation, somatic copy number alterations (SCNAs), mRNA expression, DNA methylation, and miRNA expression (Additional file 4: Table S1). These 8 cancer types include breast invasive carcinoma (BRCA), kidney renal clear cell carcinoma (KIRC), kidney renal papillary cell carcinoma (KIRP), lung adenocarcinoma (LUAD), liver hepatocellular carcinoma (LIHC), thyroid carcinoma (THCA), brain lower grade glioma (LGG) and sarcoma (SARC).

To ensure the significance of our PSM analysis results, we first performed permutation tests by randomly shifting the TERT^high^ and TERT^low^ status of samples (Additional file 2: Figure S2). By focusing on the significant feature sets in the permutation test, we identified several significantly differential molecular features (with false discovery rate < 0.05 in PSM analysis) between TERT^high^ and TERT^low^ patients across cancer types (Additional file 2: Figure S2). These molecular types exhibit distinct signature patterns across cancer types (Fig. 1b). Among them, DNA methylation and mRNA represent the most striking signatures that a number of genes display significant differences between TERT^high^ and TERT^low^ (Fig. 1b). The number of genes showing significant differences between TERT^high^ and TERT^low^ samples varied across cancer types (ranging from 176 to 9229, < 2000 genes in KIRP, KIRC and LGG, > 2000 in THCA, SARC, LIHC, LUAD and BRCA). We also observed some nondifferential features. For example, TERT^high^ and TERT^low^ showed no significant difference at somatic mutation level for KIRP, KIRC, THCA and SARC. No distinct miRNA patterns between TERT^high^ and TERT^low^ were identified for KIRP, KIRC and LGG. Taken together, these results provide a global picture of molecular differences between TERT^high^ and TERT^low^ patients. To further dissect the TERT^high^ and TERT^low^ -associated molecular mechanisms, we next performed systematic analyses at the transcriptomic (DNA methylation, mRNA expression, miRNA expression) and genomic (somatic mutation and copy number alteration patterns) levels.

### TERT^high^-specific gene expression is associated with cell cycle processes across cancers

DNA hypermethylation silences gene expression while hypomethylation leads to elevated gene expression levels [32]. We identified a large number of genes with TERT^high^ or TERT^low^-specific DNA methylation and mRNA expression [FDR < 0.05]. The number of resulting genes ranged from 52 in LGG to 4981 in THCA for DNA methylation and from 102 in KIRP to 5102 in SARC for mRNA expression. When examining the relationship between DNA methylation and mRNA expression, we found that TERT^high^ or TERT^low^-specific DNA methylation was associated with TERT^high^ or TERT^low^-specific downregulated gene expression levels in most cancer types (except for KIRC), suggesting mRNA expression regulation by DNA methylation (Additional file 5: Figure S3).

**Fig. 2:**
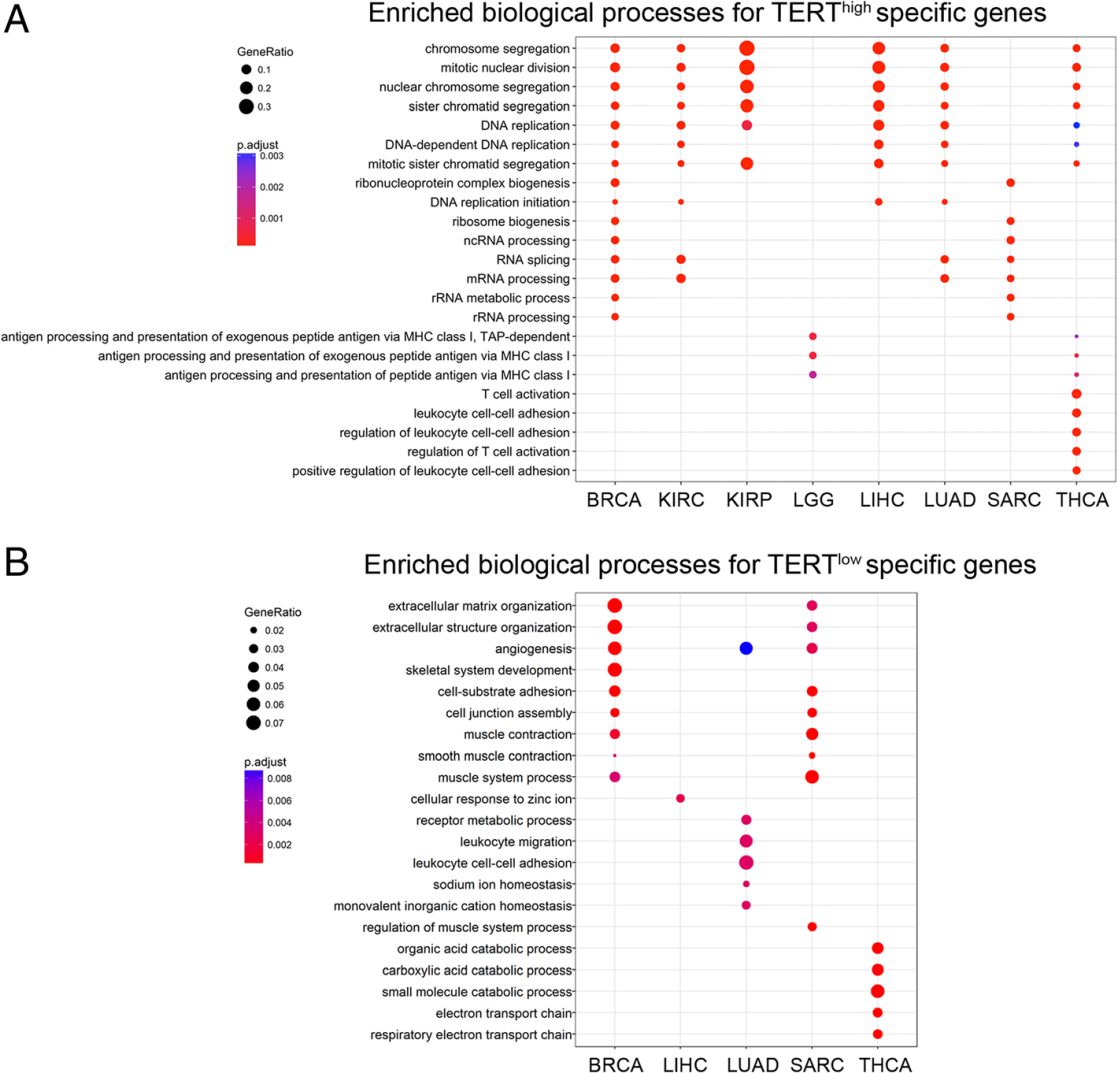
Gene ontology analysis of TERT^high^/TERT^low^-specific mRNA expression. **a** Enriched biological processes for TERT^high^-specific expressed genes across cancer types. GO terms with FDR < 0.05 are shown. **b** Enriched biological processes for TERT^low^-specific expressed genes across cancer types. GO terms with FDR < 0.05 are shown. No enriched biological processes were found for TERT^low^-specifically expressed genes in KIRP, KIRC and LGG

Notably, gene ontology enrichment analysis shows that TERT^high^-specific genes across 6 cancers (BRCA, LIHC, LUAD, THCA, KIRC and KIRP) are enriched in shared biological processes: mitotic nuclear division/DNA replication and RNA processing (Fig. 2a). In contrast, enriched biological processes of TERT^low^-specific expressing genes tend to vary across cancer types. These TERT^low^-enriched biological processes include extracellular matrix organization, angiogenesis, cell junction assembly, and muscle-related processes (Fig. 2b). These results reveal common gene signatures relevant to the cell cycle and RNA processing in TERT^high^ patients and diverse gene functions in TERT^low^ patients.

To gain additional system-level understanding of telomerase-associated mRNA expression signatures, we constructed gene coexpression networks using weighted gene coexpression network analysis (WGCNA) for each cancer type. WGCNA identified 38, 63, 76, 70, 52, 101, 47 and 58 coexpression modules in BRCA, KIRC, KIRP, LGG, LIHC, LUAD, SARC and THCA, respectively (Additional file 6: Figure S4). To examine whether the coexpression modules are invariant between TERT^high^ and TERT^low^ samples, we constructed networks using either TERT^high^ or TERT^low^ samples and performed module preservation test [33]. The results show that all modules are highly preserved across TERT^high^ and TERT^low^ networks with Zsummary > 10 for most modules, supporting the robustness and reproducibility of our identified coexpression modules (Additional file 7: Figure S5). By exploring enriched biological processes for each module by gene ontology analysis, we found four main functional module classes: (A) cell cycle/mitotic nuclear division (*P* = 1.60E-52 for BRCA, *P* = 2.59E-56 for KIRC, *P* = 1.23E-32 for KIRP, *P* = 1.08E-51 for LIHC, *P* = 1.69E-31 for LUAD, *P* = 9.35E-56 for THCA); (B) RNA splicing (*P* = 3.00E-06 for BRCA, *P* = 2.11E-07 for KIRC, *P* = 4.68E-06 for LUAD, *P* = 0.0002496 for LGG, *P* = 0.0037 for SARC); (C) extracellular matrix organization (*P* = 9.85E-39 for BRCA, *P* = 3.93E-26 for THCA) and (D) angiogenesis/regulation of vasculature development (*P* = 5.77E-17 for BRCA, *P* = 8.00E-20 for LIHC). These results further support the observation that TERT-related signatures are associated with cell cycle and RNA processing.

We also noticed that genes in the cell cycle/mitotic nuclear division module are highly expressed in TERT^high^ samples across 6 cancers (BRCA, LIHC, LUAD, THCA, KIRC and KIRP, FDR < 0.01, Additional file 8: Figure S6). Remarkably, the cell cycle/mitotic nuclear division module also showed significant enrichment of known telomere maintenance related genes Additional file 9: Table S2; *P* values range from 2.468846e-06 to 0.06327812), indicating the connectivity between cell cycle/mitotic nuclear division and telomere maintenance. Taken together, our transcriptomic analysis results demonstrate a common enriched cell cycle process in TERT^high^ patients across cancers.

### Knockdown of the hub gene TPX2 or EXO1 decreased telomerase activity and cell viability

In the gene coexpression module network, hub genes with high intramodular connectivity represent a small proportion of nodes with maximal information compared to other nodes [23]. Hub genes play important roles in the module network. Ranking the genes in the cell cycle/mitotic nuclear division module according to their connectivity measure (K), we identified several hub genes that are known to be related to telomere maintenance. For instance, one hub gene in KIRP is ASF1B (K = 35.378, ranks within the top 5 among hubs). This gene has recently been revealed to regulate epigenetic modifications at telomeres and interact with telomerase [34]. This finding implicates a putative connection between the cell cycle/mitotic nuclear division module and telomerase. To explore this further, we next examined the relationship between intramodular connectivity in the cell cycle/mitotic nuclear division module and telomerase activity. Telomerase activity was estimated by expression of a group of genes reported previously [18]. For each gene in the cell cycle/mitotic nuclear division module, we calculated the Pearson correlation between their expression levels and telomerase activity. The resulting Pearson correlation data show a significantly positive correlation between intramodular connectivity K and telomerase activity across cancer types (Pearson correlation coefficient, R > 0.4, *P* value < 2.2e-16, Additional file 10: Figure S7). These results indicate that hub genes in the cell cycle/mitotic nuclear division module are associated with telomerase activity.

**Fig. 3:**
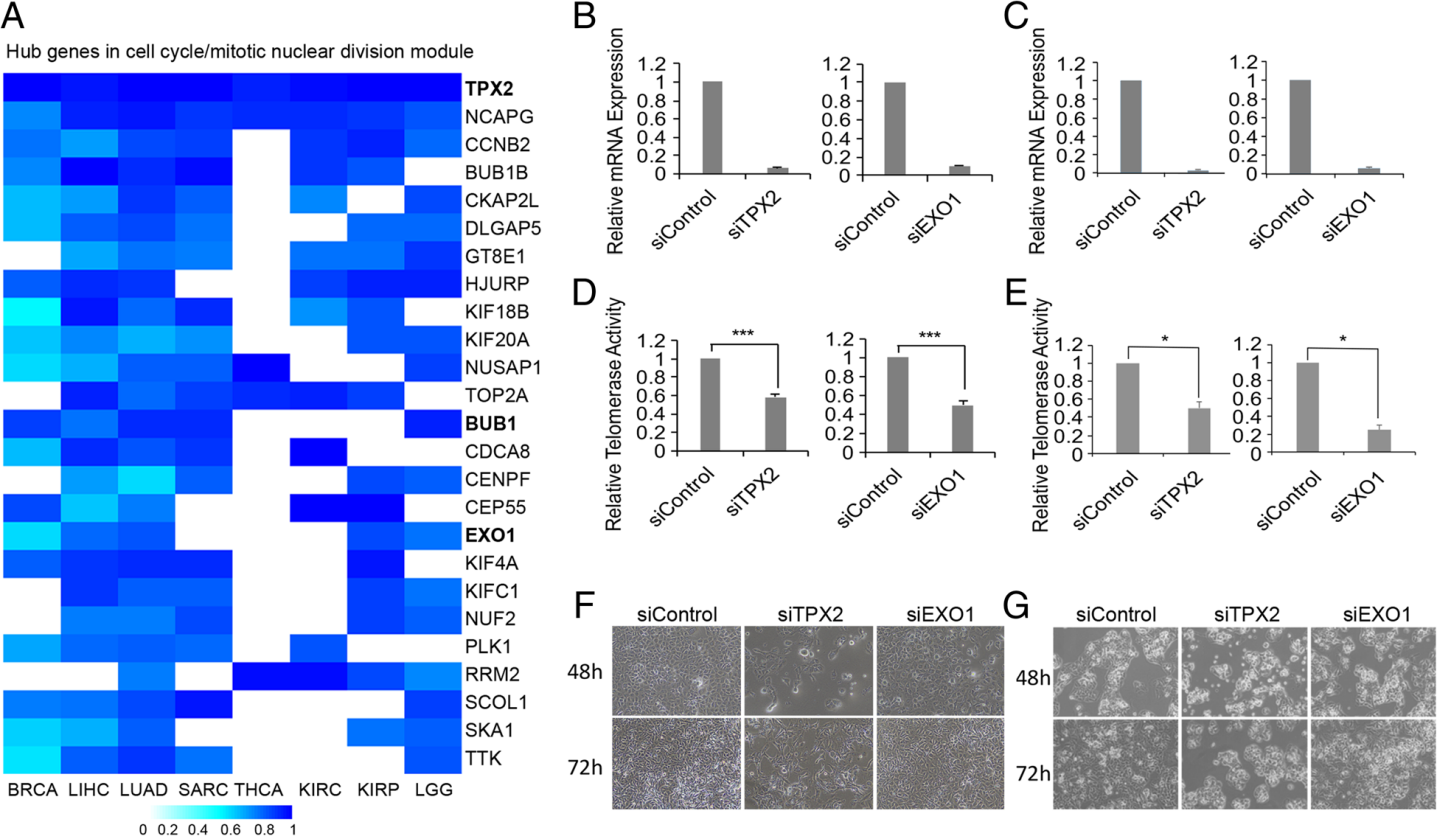
Knockdown of TPX2 or EXO1 decreases telomerase activities and cell viability. **a** Heatmaps of the degree of connectivity of genes in the cell cycle/mitotic nuclear division module, as assessed across cancers from eight different TCGA cancer types. Only hub genes exhibiting significant hub properties in at least five of the eight cancer types are shown, which we defined as consensus hub genes. TPX2, BUB1 and EXO1 are bold. **b**-**c** RT-qPCR experiments were carried out to determine the mRNA levels of endogenous TPX2 and EXO1 in HEK293T cells (**b**) and HepG2 cells (**c**) 48 h after transfection of the corresponding siRNAs. A scramble siRNA oligo was used as a control, and the results were normalized to GAPDH. **d**-**e** Relative telomerase activities were examined by Q-TRAP in TPX2- or EXO1-knockdown HEK293T cells (**d**) and HepG2 cells (**e**). Error bars indicate standard deviation, *n* = 3. ***P < 0.001. **f**-**g**) Representative images showing the states of HTC75 cells (F) and HepG2 cells (**g**) after treatment with siControl, siEXO1 or siTPX2 at 48 h and 72 h

We also observed that hub genes in the cell cycle/mitotic nuclear division module vary among cancer types (Fig. 3a). For example, TPX2, a microtubule-binding protein, is a hub gene in all 8 cancer types (Fig. 3a), while EXO1 and BUB1 are hub genes in 5 cancer types (Fig. 3a). According to the number of cancers where the gene is a hub, we defined strong (genes as hubs in at least 5 cancer types), median (genes as hubs in 4 or 3 cancer types) and weak (genes as hubs in 2 or 1 cancer type) hub gene groups (Additional file 3: Table S3). We reasoned that our analysis would identify potential telomerase regulators, and genes in the strong hub group would have a higher degree of association with telomerase activity compared with those in other groups. To test this hypothesis, we randomly selected genes from strong- and median hub groups (TPX2 and EXO1 in strong group, FOXM1 and RAD54L in median group) and non-hub cell cycle gene NEIL3 (as negative control) for experimental validation. We knocked down these genes individually in HEK293T and liver hepatocellular carcinoma HepG2 cells. The siRNA oligo achieved approximately 80% knockdown efficacy of mRNA expression compared to the siRNA control (Fig. 3 b and c, Additional file 11: Figure S8A-C). Intriguingly, knockdown of the strong hub group gene TPX2 or EXO1 significantly reduced 50% telomerase activity in both cells (Fig. 3 d and e). Knockdown of the median hub group gene resulted in less telomerase activity (~ 40% decrease in RAD54L knocked down HEK293T cells, Additional file 11: Figure S8D) or increased telomerase activity (RAD54L knocked down HepG2 cells in Additional file 11: Figure S8D and FOXM1 knocked down cells in Additional file 11: Figure S8E). As expected, the reduction in the negative control non-hub cell cycle gene NEIL3 did not change any telomerase activity (Additional file 11: Figure S8F). In parallel with decreased telomerase activities, TPX2 or EXO1 knockdown reduced the viability of fibrosarcoma HTC75 cells and HepG2 cells (Fig. 3f). These data suggest that the hub genes TPX2 and EXO1 may regulate telomerase activity and that a strong hub group contains potential telomerase regulators.

### The TERT^high^-specific miR-17-92 cluster is associated with telomere shortening

**Fig. 4:**
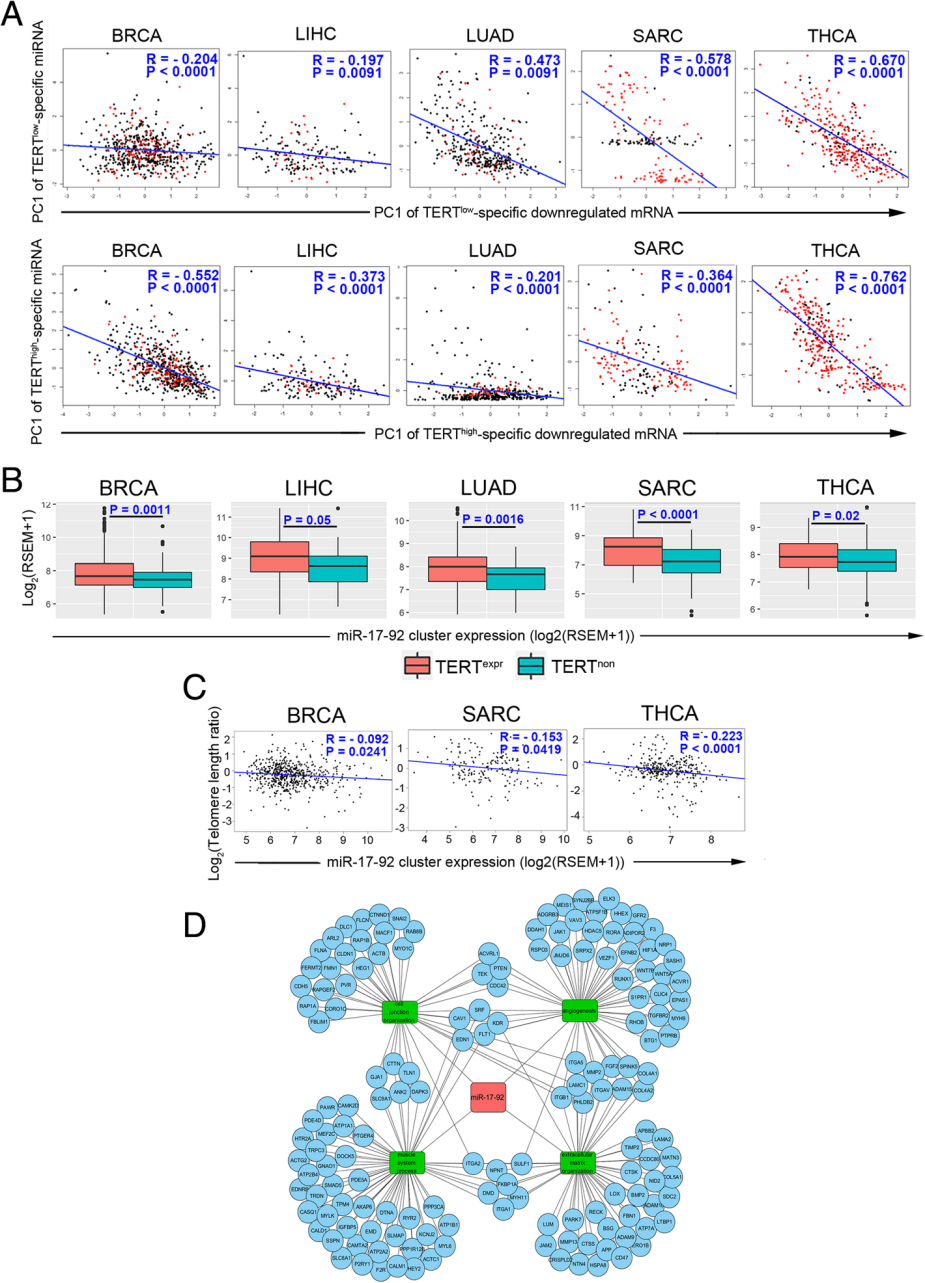
The TERT^high^-specific miR-17-92 cluster is associated with telomere shortening. **a** Correlations between the PC1s of expression levels of TERT^high^/TERT^low^-specific miRNAs and TERT^high^/TERT^low^-specific mRNAs that are predicted targets. Pearson correlation coefficients (R) and *P* values are shown. **b** Box plots show higher expression levels of the miR-17-92 cluster in TERT^high^ patients compared to TERT^low^ patients. miR-17-92 cluster expression is defined as the mean of the expression of miRNAs in this cluster. The expression value is represented as log2 transformed (RSEM+ 1). P values between two groups are shown, which were calculated by the Wilcoxon rank sum test. **c** Correlation plots show a negative correlation between matching tumor/normal telomere length ratios and expression of the miR-17-92 cluster. Pearson correlation coefficient R and P values are shown. **d** Network of the miR-17-92 cluster, its target mRNAs and enriched biological processes for target mRNAs

miRNA plays a critical role in the posttranscriptional regulation of gene expression [35]. In five of eight cancer types (BRCA, LIHC, LUAD, SARC and THCA, Fig. 1b), we were able to detect several TERT^high^ or TERT^low^-specific-expressing miRNAs [FDR < 0.05], ranging from 21 in THCA to 243 in SARC. Assessing the correlation between the first principle component of TERT^high^ or TERT^low^-specific expressing miRNAs and those of their predicted TERT^high^ or TERT^low^-specific downregulated mRNA targets. We observed a significantly negative correlation in 5 cancer types (Fig. 4a). This result suggests that TERT^high^/TERT^low^-specific mRNAs are regulated by TERT^high^/TERT^low^-specific miRNAs.

A recent study has reported that miRNA miR-19b augments TERT expression by targeting PITX1 [36]. miR-19b is transcribed from the miR-17-92 cluster, which comprises miR-17, miR-18a, miR-19a, miR-20a, miR-19b-1 and miR-92a-1 [37]. In the five cancer types with TERT^high^ or TERT^low^-specific-expressing miRNAs (BRCA, LIHC, LUAD, SARC and THCA), we found that the expression of the miR-17-92 cluster is TERT^high^-specific (Fig. 4b). Interestingly, the expression level of the miR-17-92 cluster was negatively correlated with the tumor/normal telomere length ratio in TERT^high^ patients in BRCA, SARC and THCA (Fig. 4c). Many targets of the TERT^high^-specific miR-17-92 cluster belong to multiple extracellular matrix organization-related biological processes that are enriched in TERT^low^-specific cancers (Fig. 4d). These results indicate that the miR-17-92 cluster is associated with telomere shortening in TERT^high^ patients in certain cancers and that the miR-17-92 cluster may maintain telomerase activity by suppressing extracellular matrix organization genes.

### TERT^high^-specific somatic mutations and copy-number alterations

**Fig. 5:**
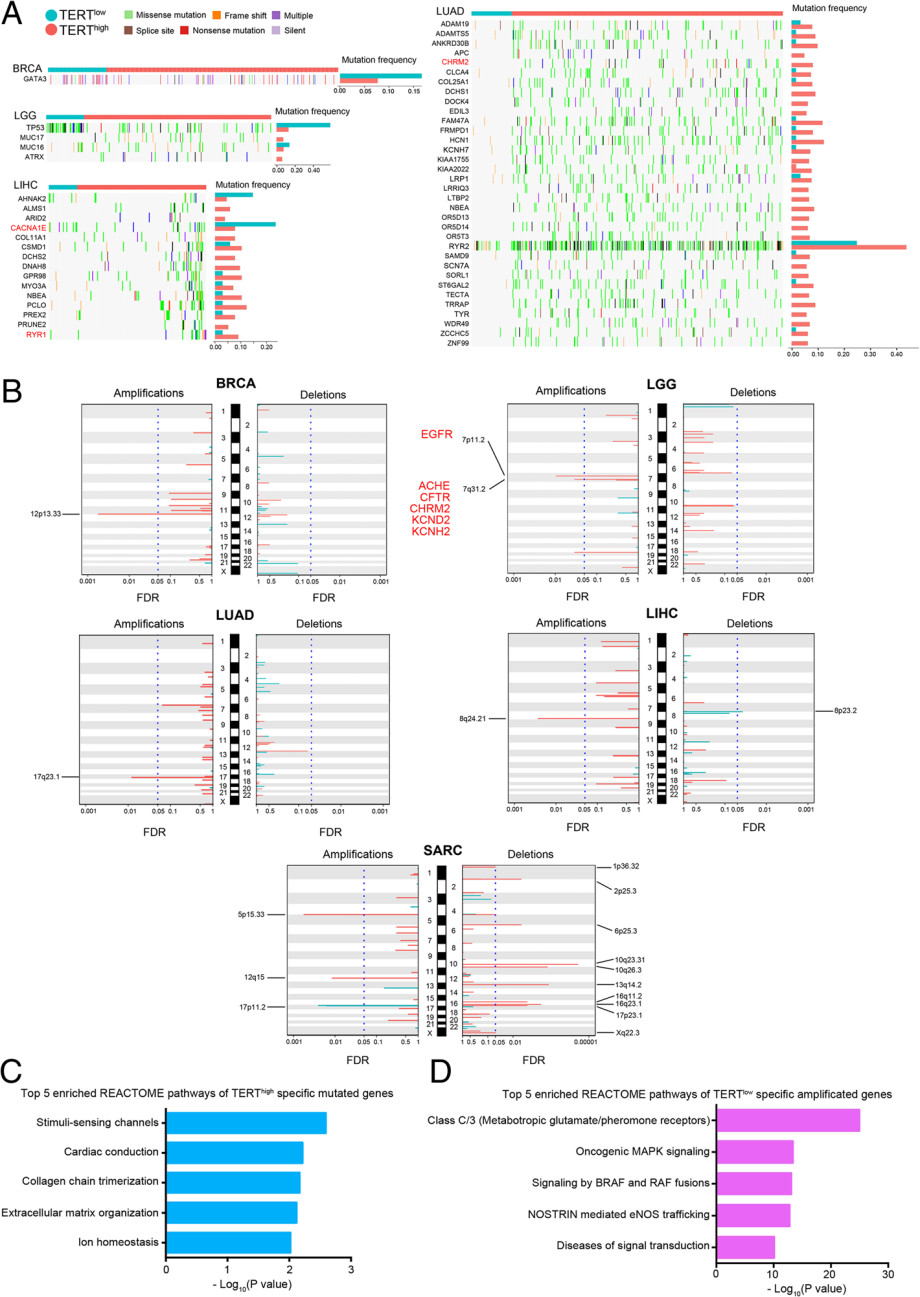
TERT^high^/TERT^low^ -specific somatic mutation and somatic copy number alteration signatures. **a** Overview of genes with TERT^high^/TERT^low^ -specific mutation signatures in LGG, LIHC and LUAD (FDR < 0.05). Samples are displayed as columns with the TERT^high^/TERT^low^ label on the top, and different colors indicate different types of somatic mutations. The bar plots show the recalibrated mutation frequencies after propensity score weighting. **b** The genome-wide, TERT^high^/TERT^low^-specific focal amplification/deletion patterns in BRCA, LGG, LUAD, LIHC and SARC. The TERT^low^-specific SCNA peaks are shown in turquoise, and the TERT^high^-specific SCNA peaks are shown in tomato. The significant SCNA regions (FDR < 0.05) are indicated by the vertical blue dotted lines. The targets of FDA-approved drugs are highlighted in red. **c** Bar plot showing the top 5 enriched REACTOME pathways of TERT^high^-specific mutated genes. Enrichment is represented as –log_10_(P value). **d** Bar plot showing the top 5 enriched REACTOME pathways of TERT^high^-specific amplified genes. Enrichment is represented as –log_10_(P value)

We next investigated differences between TERT^high^ and TERT^low^ mechanisms at the genomic level. By setting the FDR = 0.05 and focusing on highly mutated genes (≥ 5% mutation frequency), we identified 1 TERT^low^-specific somatic mutation gene in BRCA, 2 TERT^low^-specific and 2 TERT^high^-specific somatic mutation genes in LGG, 2 TERT^low^-specific and 13 TERT^high^-specific genes in LIHC, 34 TERT^high^-specific genes in LUAD (Fig. 5a).

To characterize TERT^high^/TERT^low^-specific SCNAs, we analyzed the most significant SCNAs (including both focal and arm-level amplifications/deletions) identified by GISTIC [38]. At FDR = 0.5, we found significant TERT^high^/TERT^low^-specific SCNAs with 8 amplification peaks (12p13.33 in BRCA; 7p11.2 and 7q31.2 in LGG; 17q23.1 in LUAD; 8q24.21 in LIHC; 5p15.33, 12q15 and 17p11.2 in SARC) and 11 deletion peaks (8p23.2 in LIHC; 1p36.32, 2p25.3, 6p25.3, 10q23.31, 10q26.3, 13q14.2, 16q11.2, 16q23.1, 17p23.1 and Xq22.3 in SARC) (Fig. 5b). Consistent with previous findings that TERT^low^ cases contain fewer copy number segments [18], most of these significant amplification/deletion peaks are TERT^high^-specific (8 amplification peaks except 17p11.2 and 10 deletion peaks except 8p23.2), whereas only 1 amplification peak in SARC and 1 deletion peak in LIHC are TERT^low^-specific (Fig. 5b).

To understand the mechanism underlying TERT^high^-specific mutation and amplification, we next examined enriched pathways of TERT^high^-specific mutated or amplified genes. REACTOME pathway analysis showed that TERT^high^-specific mutated genes were enriched in stimuli-sensing channels, cardiac conduction and extracellular matrix organization (Fig. 5c). Extracellular matrix organization was found to be enriched for TERT^low^-specific mRNA genes (Fig. 2b), suggesting that TERT^high^ cancers may silence genes whose expression is high in TERT^low^ cancers. In addition, REACTOME pathway analysis showed that TERT^high^-specific amplified genes are enriched in Class C/3, oncogenic MAPK signaling (Fig. 5d). Given that oncogenic MAPK signaling can promote cell cycle progression [39], these data indicate that TERT^high^ cancers can amplify growth signaling genes to maintain cell proliferation.

### TERT-associated mutation/SCNAs and mRNA expression signatures contain clinically actionable targets

To investigate the clinical implications of the TERT-associated mutation/SCNAs and mRNA expression signatures, we searched for targets of FDA-approved drugs in the signatures. To identify highly confident targets, we selected targets that met one of the following criteria: 1) targets that were mutated or amplified or 2) targets that were consistently upregulated at least 2-fold in TERT^high^ or TERT^low^ samples compared to adjacent normal samples across cancer types.

**Fig. 6:**
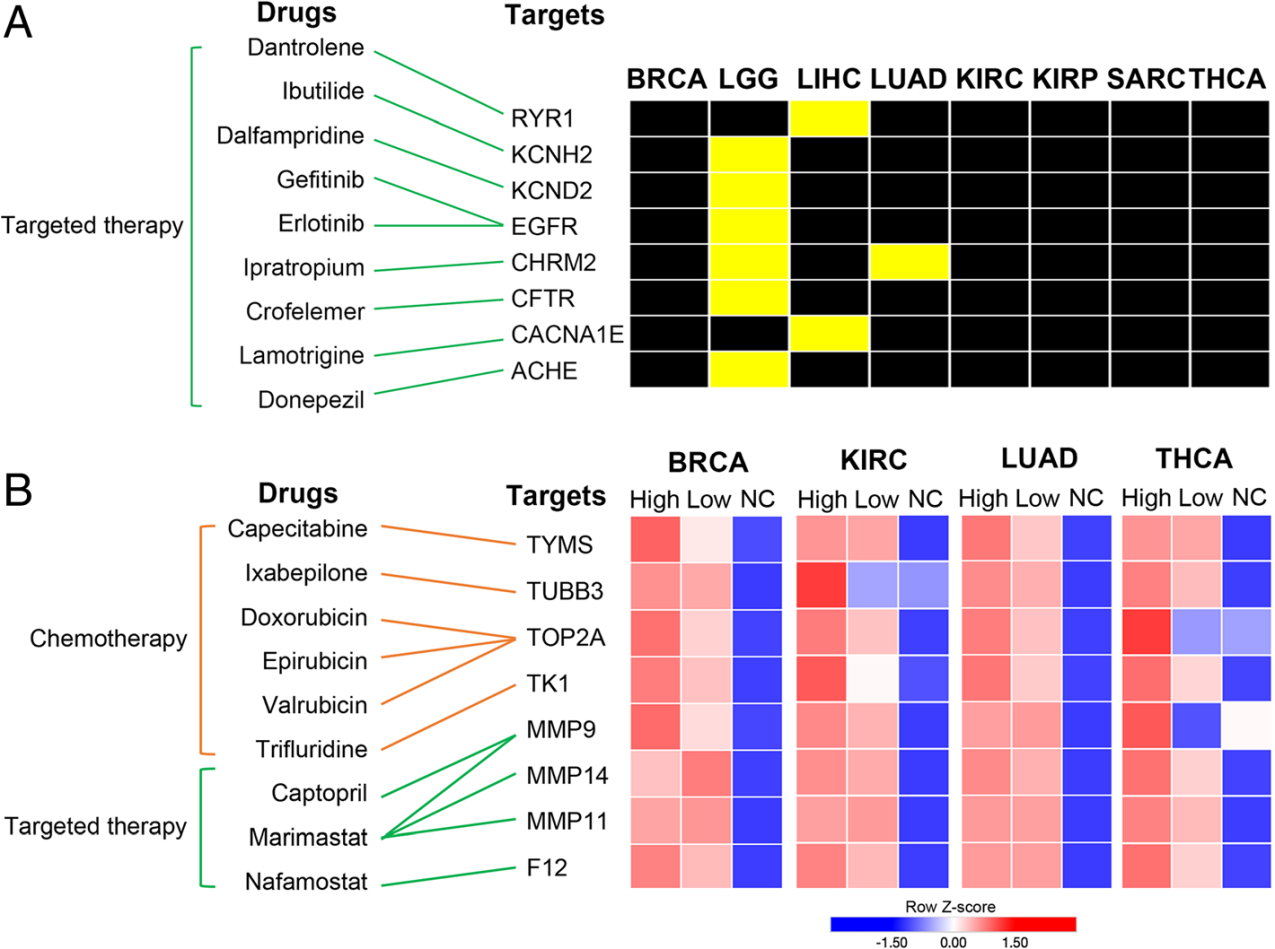
TERT-associated signatures harbor clinically actionable genes. **a** The mapping between FDA-approved drugs and their related clinically actionable genes (left) across cancer types (right). Yellow boxes indicate cancers with mutated genes, while black boxes indicate cancers without mutated genes. **b** The mapping between FDA-approved drugs and their related clinically actionable genes (left) and their average mRNA expression in cancer and adjacent normal samples across cancer types (right). High, TERT high group; Low, TERT low group; NC, adjacent normal sample group. Red indicates relatively high expression, while blue indicates relatively low expression

Examining the TERT-associated mutation/SCNA list, we found 8 drug targets with mutations/SCNAs across cancer types (Fig. 6a). All targets are mutated or amplified in either LGG, LIHC or LUAD but not in other cancers. LGG has the highest number of amplified drug targets (6 out of 8, yellow colored boxes) (Fig. 6a). In addition, TERT-associated mRNA expression signatures contain targets of FDA-approved drugs. In this analysis, we focused on those cancers (BRCA, KIRC, LUAD and THCA) with adjacent normal samples available. Eight targets were identified based on the criteria that they had at least 2-fold upregulation in cancer (Fig. 6b). Drugs for these targets can be categorized into 2 groups: chemotherapy and targeted therapy. Many drugs have been widely used in the clinic. In targeted therapy, gefitinib and erlotinib, EGFR inhibitors, were approved for the treatment of cancers with EGFR mutations or hyperactivation, including certain breast and lung cancers [40, 41]. In chemotherapy, the anthracycline drugs doxorubicin and epirubicin are used in the treatment of multiple cancers [42].

### Identification of two cancer subtypes with distinct telomerase activities and survival rates based on TERT-associated multi-omics molecular signatures

**Fig. 7:**
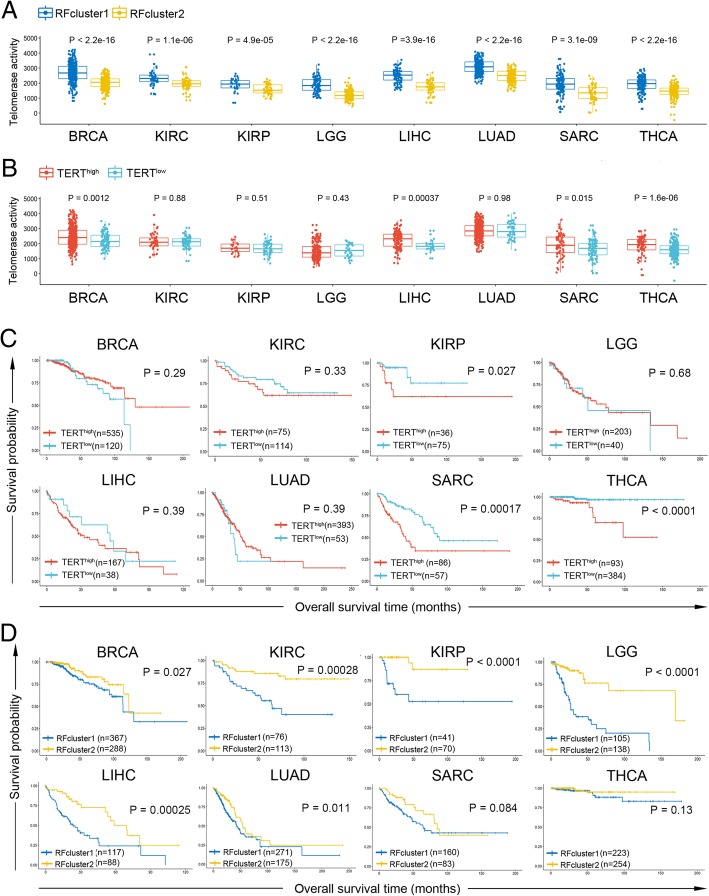
Genomic and transcriptomic marker-guided random forest clustering identified two cancer subtypes with distinct telomerase activity and survival rates. **a** Box plots show differences in estimated telomerase activity between RFcluster1 and RFcluster2 groups. The P value was calculated by the Wilcoxon rank sum test. **b** Box plots show differences in estimated telomerase activity between the TERT^high^ and TERT^low^ groups. The P value was calculated by the Wilcoxon rank sum test. **c** Kaplan-Meier plots show overall survival rates for the TERT^high^ and TERT^low^ groups. The P value was calculated using the log-rank test. **d** Kaplan-Meier plots show overall survival rates for the RFcluster1 and RFcluster2 groups. The P value was calculated using the log-rank test

In an attempt to examine the power and utility of these TERT-associated signatures for cancer subtype classification and prognostic analysis, we developed a multi-omics random forest classifier using the following signatures as guides: (1) “global markers”, which consist of consensus hub genes of cell cycle/mitosis nuclear division module in WGCNA (Additional file 3: Table S3) and miR-17-92 cluster, and (2) “cancer-specific markers”, which contain TERT^high^/TERT^low^-specific top 20 expressed genes (including TERT) and DNA methylation probes, and the top 5 expressed miRNAs, SNPs and SCNAs. With this classifier, we were able to identify two subtypes (RFcluster1 and RFcluster2) for each cancer type. RFcluster1 showed higher levels of telomerase activity (Fig. 7a). These two subtypes show more significant differences in telomerase activity when compared with the TERT^high^ and TERT^low^ groups (Fig. 7 a and b). Furthermore, these two subtypes showed significant differences in the overall survival rate in 6 cancer types except for SARC and THCA (Fig. 7d), whereas the TERT^high^ and TERT^low^ groups showed differences in only 3 cancer types (KIRP, SARC and THCA) (Fig. 7c) (see also Additional file 12: Table S4 for Cox regression results). Comparison among different predictors in the prediction of patient survival showed that the multi-omics random forest predictor outperformed predictors using TERT expression or telomerase activity alone (Additional file 12: Table S4). This demonstrates that integrating multi-omics molecular information associated with TERT yields two cancer subtypes with more discrimination in telomerase activity and patient survival compared with those using TERT^high^ and TERT^low^ information only.

## Discussion

By applying a propensity score matching algorithm to control potential confounder effects, we comprehensively characterized the molecular differences between TERT^high^ and TERT^low^ patients across 8 cancer types. Our results reveal large diverse molecular signatures and common pathways, such as the cell cycle, at the transcriptomic level. By combining TERT^high^ and TERT^low^-specific genomic and transcriptomic differences, we developed a multi-omics random forest classifier. This classifier successfully identified two groups of patients with different telomerase activities and overall survival rates, providing novel insights that link telomerase-related signatures to patient survival.

Compared to TERT^low^ patients, TERT^high^ patients display higher mRNA expression levels of cell cycle genes and lower expression of genes in extracellular matrix organization and angiogenesis. Moreover, TERT^high^ patients tend to have mutated genes enriched in extracellular matrix organization and amplify genes in cell cycle progression-related MAPK signaling. Consistent with our finding, a recent report has found that cell cycle genes were upregulated, whereas extracellular matrix production and angiogenesis genes were downregulated in primary BJ fibroblasts when they were immortalized by overexpression of human TERT in vitro [43]. We also noticed that RNA processing-related processes rank second among TERT^high^-specific biological processes. In line with this finding, recent studies have shown that telomerase promotes cell proliferation by augmenting tRNA expression and stimulating ribosomal DNA transcription [44, 45]. Taken together, these data indicate that maintenance of telomerase activity may require high expression of cell cycle-related genes and that TERT may promote unlimited cell proliferation by reducing extracellular matrix production and enhancing translation in a noncanonical manner. These data also demonstrate that telomerase or TERT is an essential factor in cell survival and growth regulatory mechanisms in which telomere maintenance, protein synthesis and cell migration are involved.

Our analysis also revealed that the expression of the TERT^high^-specific miR-17-92 cluster negatively correlates with the tumor/normal telomere length ratio. These data suggest that telomere attrition and TERT activation may promote miR-17-92 cluster expression in certain cancer types. Targets of the TERT^high^-specific miR-17-92 cluster are involved in multiple biological processes, including extracellular matrix organization, angiogenesis, cell junction organization and muscle system process, indicating that miR-17-92 might assist in the maintenance of telomerase activity and telomere by inhibiting these processes.

Our coexpression network analysis identified a cell cycle/mitotic nuclear division module linked to telomerase activity regulation. In particular, TPX2 and EXO1, two strong hub genes, are potential telomerase regulators. Knockdown of TPX2 or EXO1 significantly decreased telomerase activity. EXO1 is a DNA exonuclease and resects telomere ends [46], while TPX2 has not been implicated in telomere biology. Future work will dissect the detailed mechanisms of TPX2 and EXO1 in the regulation of telomerase activity. Moreover, some hub genes in the cell cycle/mitotic nuclear division module have been shown to play important roles in telomere regulation. For example, BUB1 directly phosphorylates TRF1 and promotes telomere replication [47]. Future studies will understand the roles of hub genes in telomere regulation and noncanonical functions of telomerase.

Given the central role in cellular immortality, telomerase has garnered significant attention as an anticancer drug target [48]. Numerous telomerase inhibitors have been designed over the past decades, some of which have successfully passed stage I in clinical trials [48]. However, anti-telomerase therapies have been shown to induce ALT in mouse and human cancer cells [49]. Therefore, our findings of FDA-approved drug targets for TERT^high^ cancer may provide alternative options for treating this type of cancer.

This study has limitations. We recognize that stratification of patients by cancer stage and TERT promoter mutation has not been conducted. TERT promoter mutations have recently been shown to be major drivers of TERT expression. In our preliminary analysis of LGG, THCA and LIHC patients, we found that the multi-omics signature performs better than the TERT promoter mutation in the classification of TERT^high^ and TERT^low^ patients for THCA and LIHC and that the combination of both parameters achieves the highest performance (data not shown). Validations in other cancer types and a large cohort of cancer patients are warranted in the future.

## Conclusions

In summary, our report of molecular differences between TERT^high^ and TERT^low^ cancers provides essential insights into telomerase-associated alterations in cancer and opens new avenues for treating cancer.

## Additional files

Additional file 1: **Figure S1.** Potential confounders surveyed and balanced. (TIF 2290 kb)

Additional file 2: **Figure S2.** Assessment the robustness of molecular signatures. (TIF 3967 kb)

Additional file 3: **Table S3.** Hub genes of cell cycle/mitotic nuclear division module across eight cancers. (DOCX 102 kb)

Additional file 4: **Table S1.** Number of cancer cases analyzed in this study (DOCX 17 kb)

Additional file 5: **Figure S3.** Regulation of mRNA expression by DNA methylation. (TIF 815 kb)

Additional file 6: **Figure S4.** Coexpression modules in 8 cancers. (TIF 11429 kb)

Additional file 7: **Figure S5.** Preservation of mRNA co-expression modules. (TIF 2988 kb)

Additional file 8: **Figure S6.** Genes in cell cycle/mitotic nuclear division module identified by WGCNA are highly expressed in TERT^high^ samples across 6 cancers. (TIF 1270 kb)

Additional file 9: **Table S2.** Enrichment of validated telomere maintenance related Genes within the cell cycle/mitotic nuclear division module genes. (DOCX 14 kb)

Additional file 10: **Figure S7.** Association analysis between the cell cycle/mitosis module and telomerase activity. (TIF 1280 kb)

Additional file 11: **Figure S8.** Telomerase activity changes of RAD54L, FOXM1 or NEIL3 knockdown. (TIF 917 kb)

Additional file 12: **Table S4.** Summary of cox regression results. (DOCX 14 kb)

## Data Availability

The datasets used and/or analysed during the current study are available from the corresponding author on reasonable request.

## Abbreviations

ALT: Alternative lengthening of telomeres
BRCA: Breast invasive carcinoma
FDA: Food and drug administration
KIRC: Kidney renal clear cell carcinoma
KIRP: Kidney renal papillary cell carcinoma
LGG: Brain lower grade glioma
LIHC: Liver hepatocellular carcinoma
LUAD: Lung adenocarcinoma
MAPK: Mitogen-activated protein kinase
PSM: Propensity score matching
Q-TRAP: Real-time quantitative PCR-based TRAP
SARC: Sarcoma
SCNA: Somatic copy-number alternations
TCGA: The Cancer Genome Atlas
TERT: Telomerase reverse transcriptase
THCA: Thyroid carcinoma
WGCNA: Weight gene coexpression network analysis
