# Supplementary figures and images for: Pan-cancer analysis identifies telomerase-associated signatures and cancer subtypes
Source: Mol Cancer. 2019 Jun 10;18:106. doi: 10.1186/s12943-019-1035-x (PMC6556968; doi:10.1186/s12943-019-1035-x)

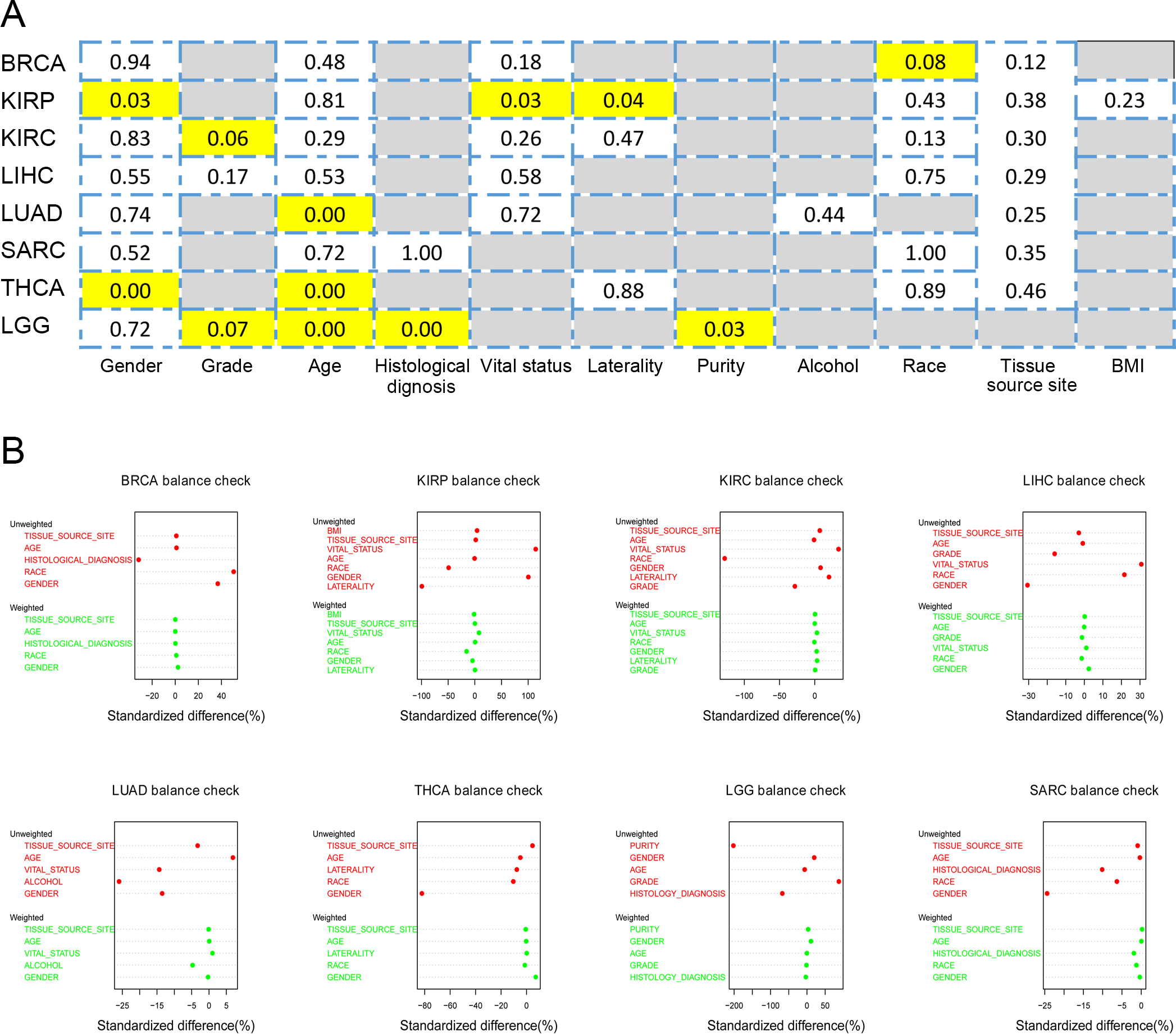

Supplement: Supplementary file 1 — Figure S1. Potential confounders surveyed and balanced. (TIF 2290 kb) [file 12943_2019_1035_MOESM1_ESM.tif]

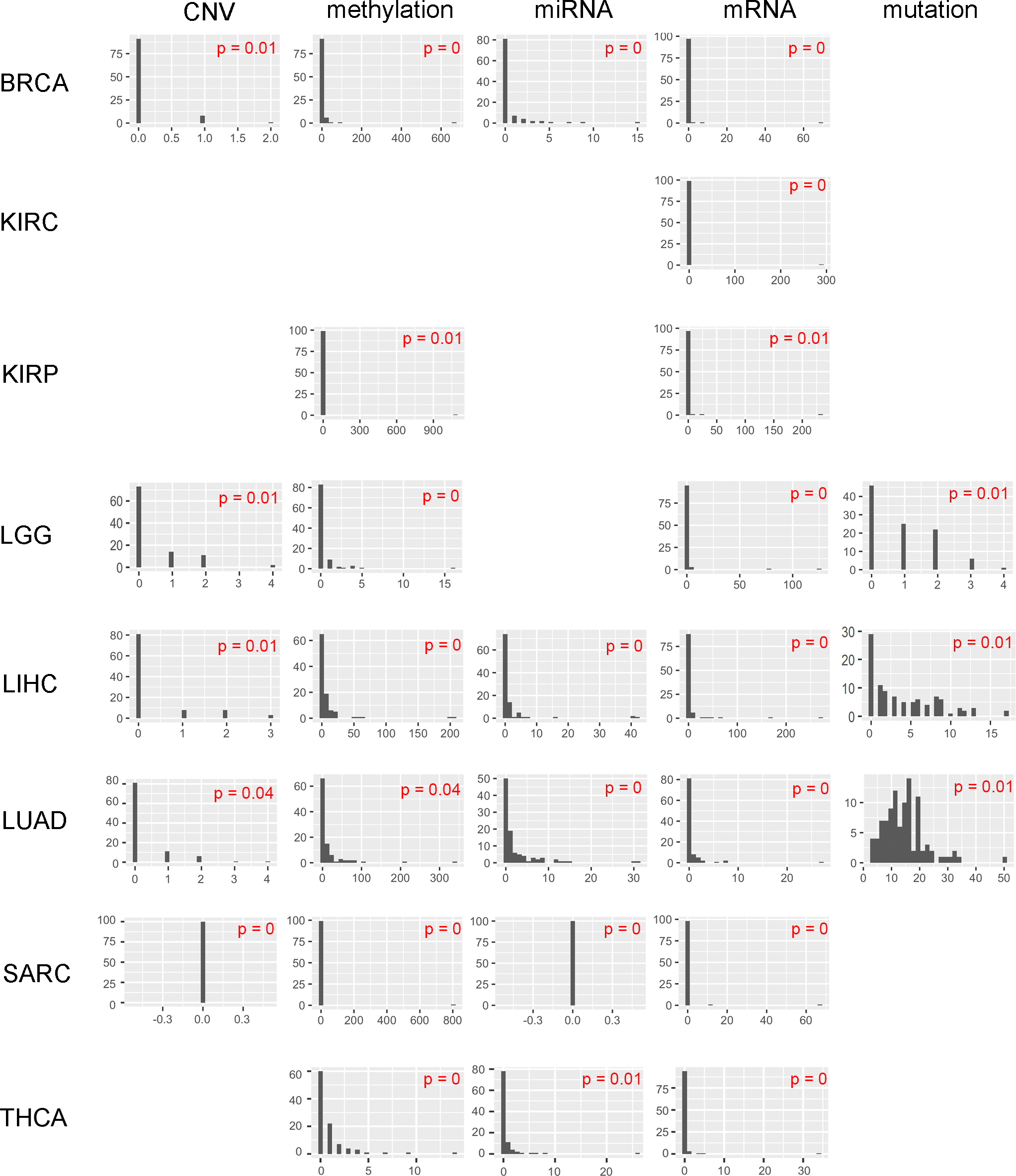

Supplement: Supplementary file 2 — Figure S2. Assessment the robustness of molecular signatures. (TIF 3967 kb) [file 12943_2019_1035_MOESM2_ESM.tif]

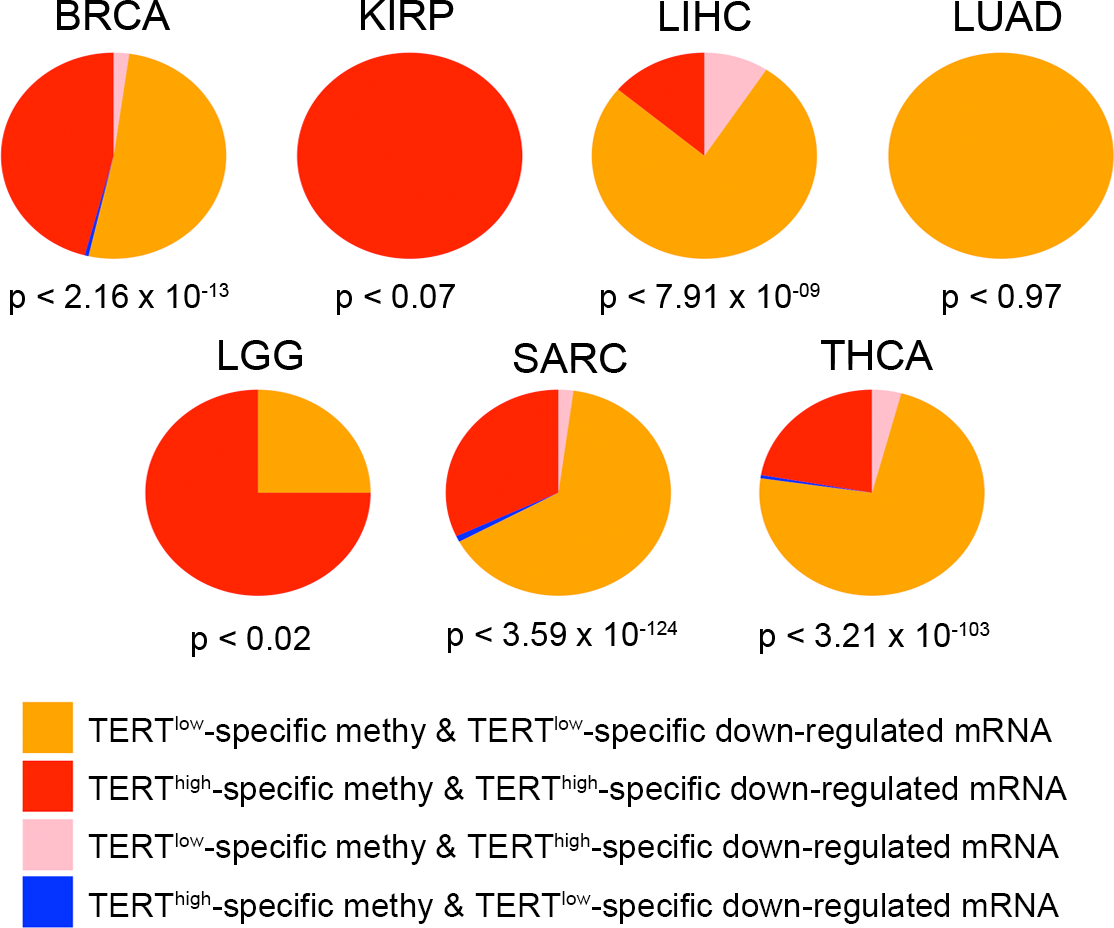

Supplement: Supplementary file 5 — Figure S3. Regulation of mRNA expression by DNA methylation. (TIF 815 kb) [file 12943_2019_1035_MOESM5_ESM.tif]

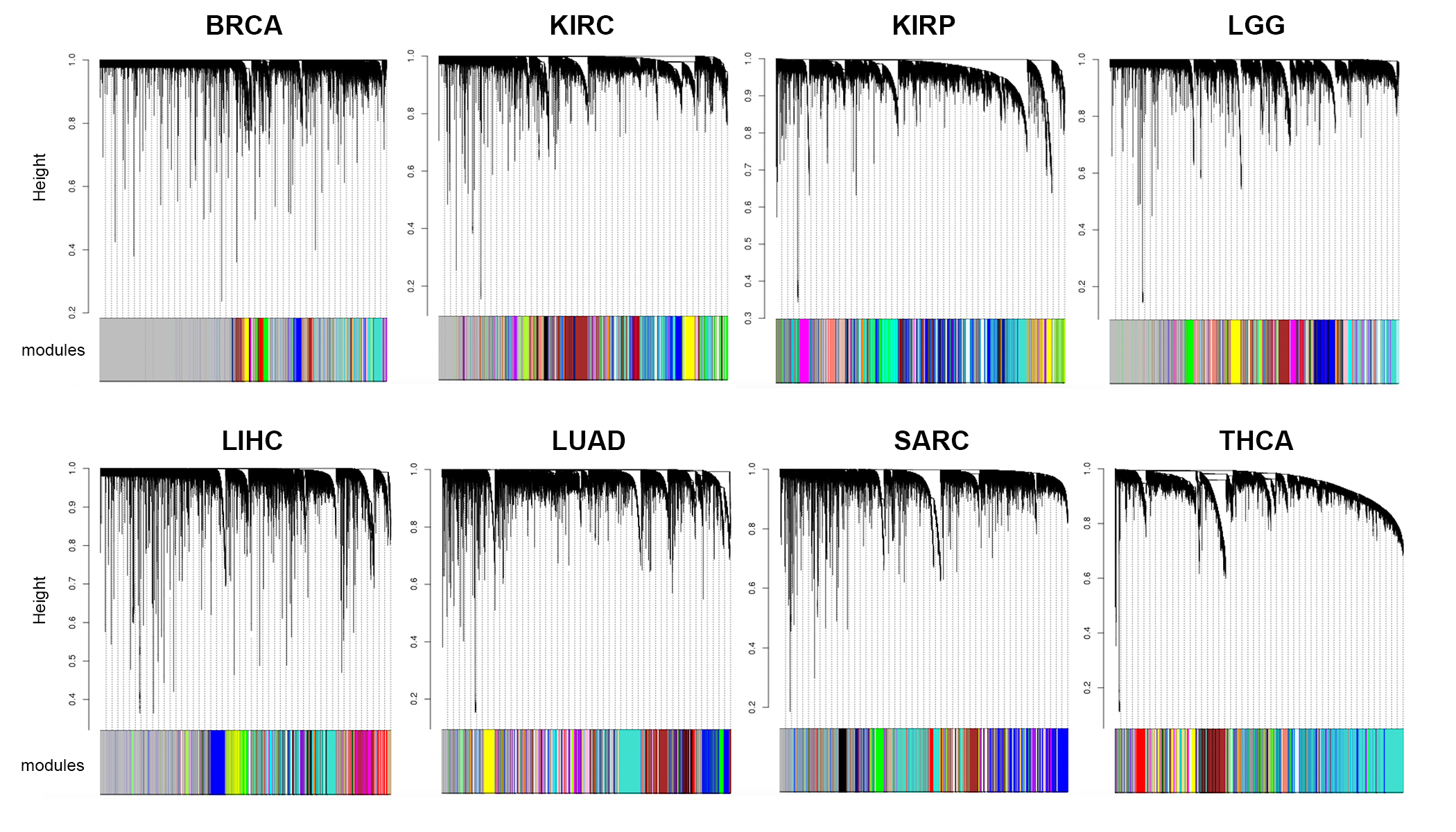

Supplement: Supplementary file 6 — Figure S4. Coexpression modules in 8 cancers. (TIF 11429 kb) [file 12943_2019_1035_MOESM6_ESM.tif]

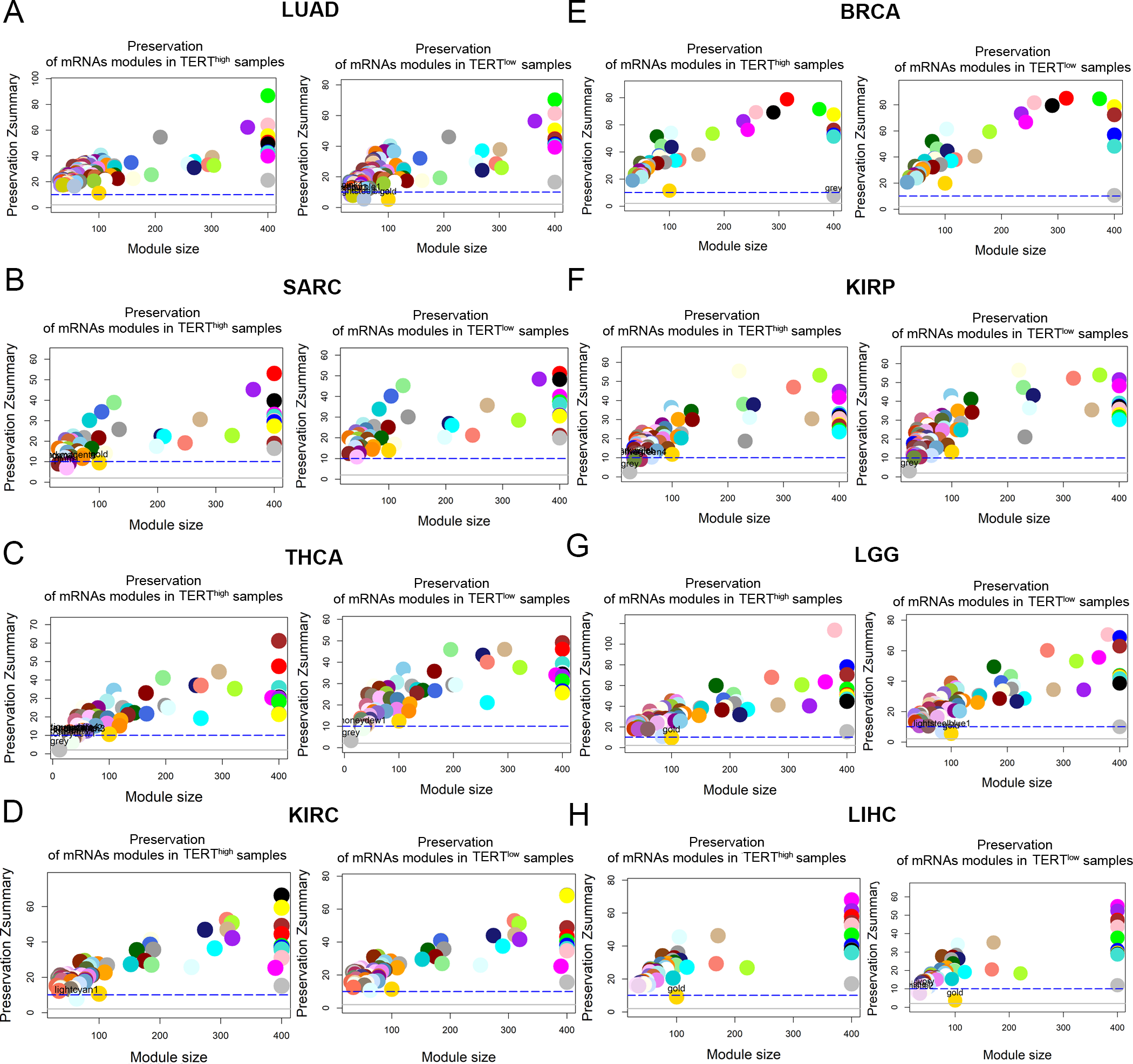

Supplement: Supplementary file 7 — Figure S5. Preservation of mRNA co-expression modules. (TIF 2988 kb) [file 12943_2019_1035_MOESM7_ESM.tif]

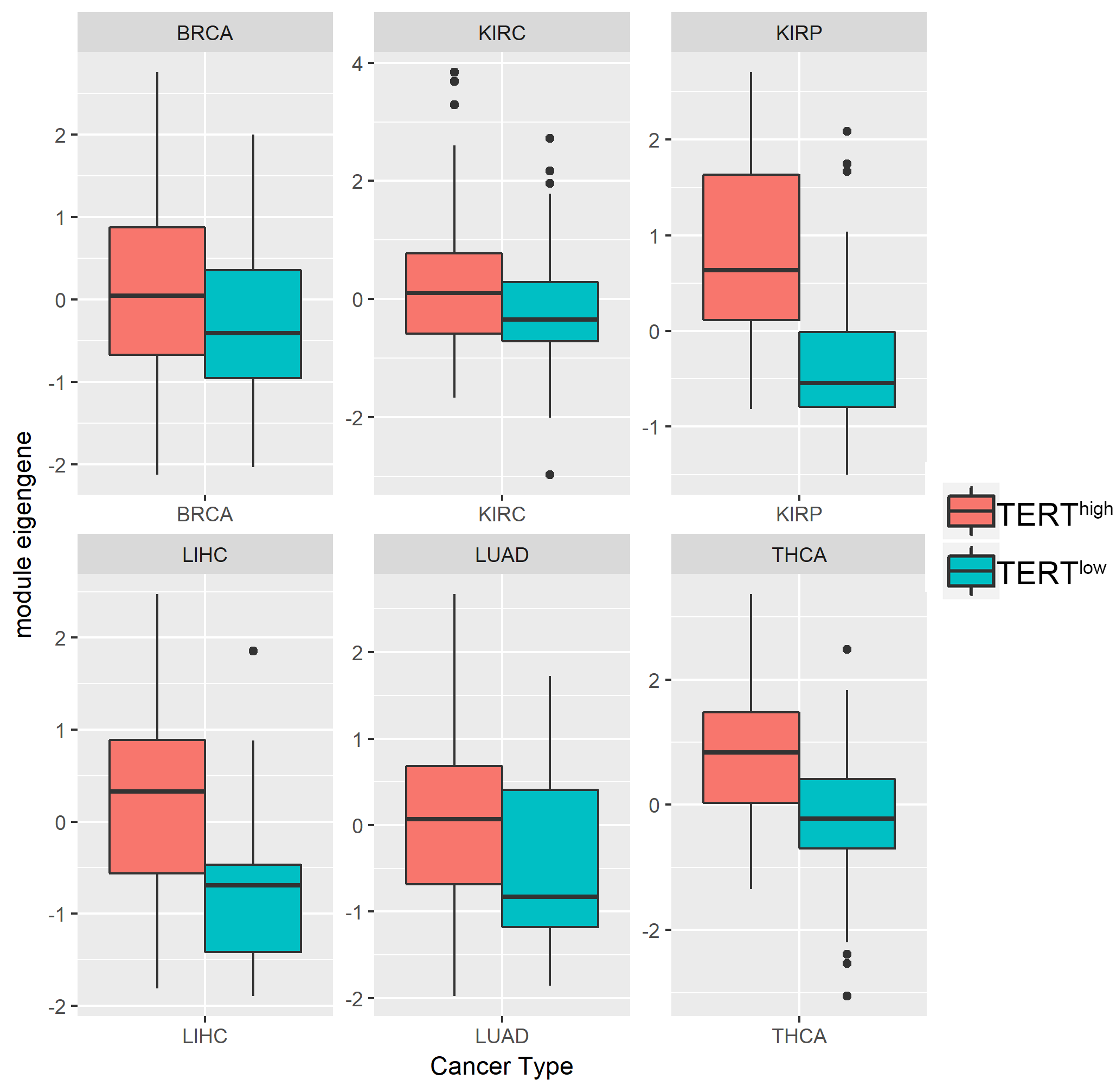

Supplement: Supplementary file 8 — Figure S6. Genes in cell cycle/mitotic nuclear division module identified by WGCNA are highly expressed in TERThigh samples across 6 cancers. (TIF 1270 kb) [file 12943_2019_1035_MOESM8_ESM.tif]

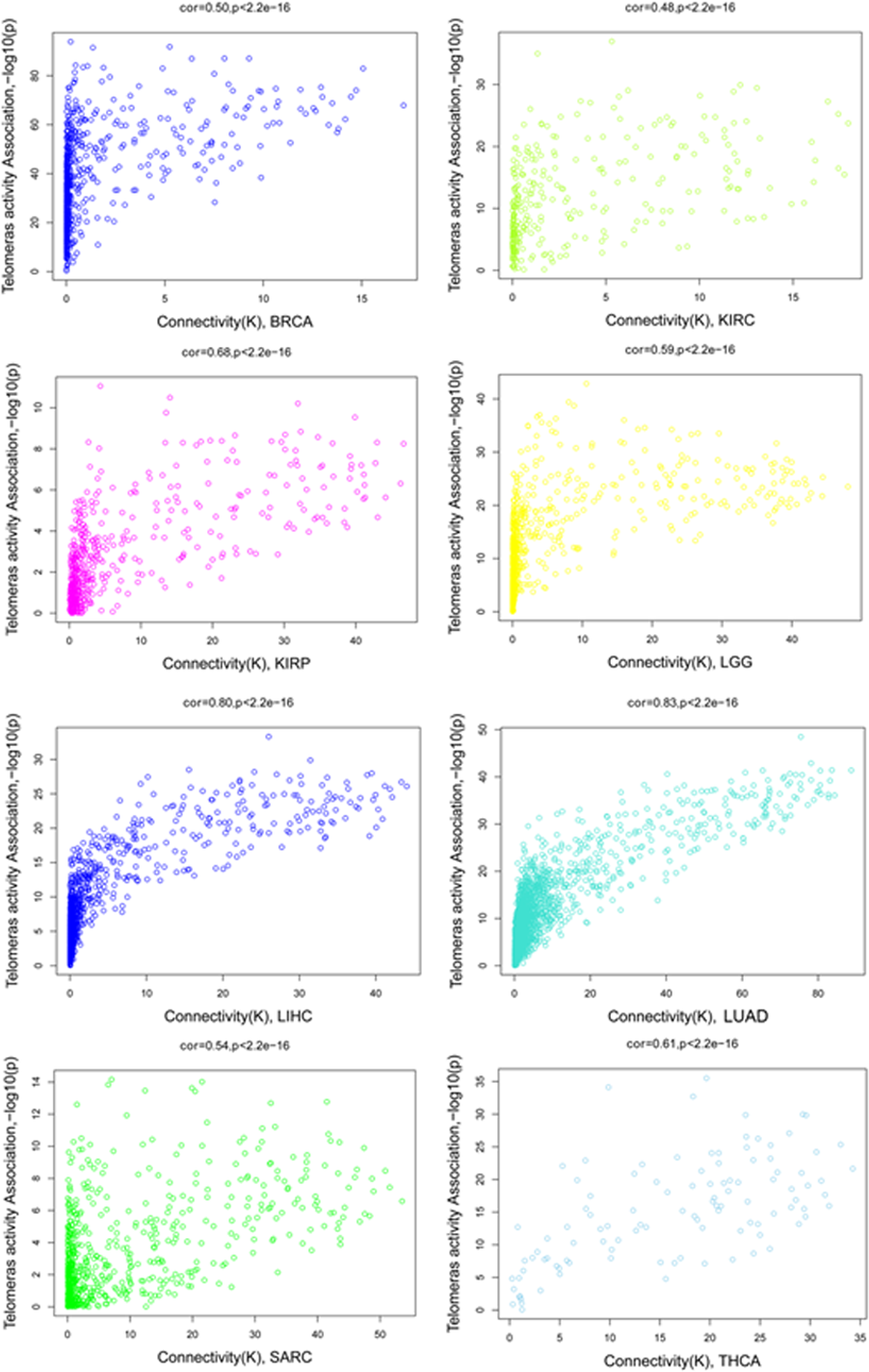

Supplement: Supplementary file 10 — Figure S7. Association analysis between the cell cycle/mitosis module and telomerase activity. (TIF 1280 kb) [file 12943_2019_1035_MOESM10_ESM.tif]

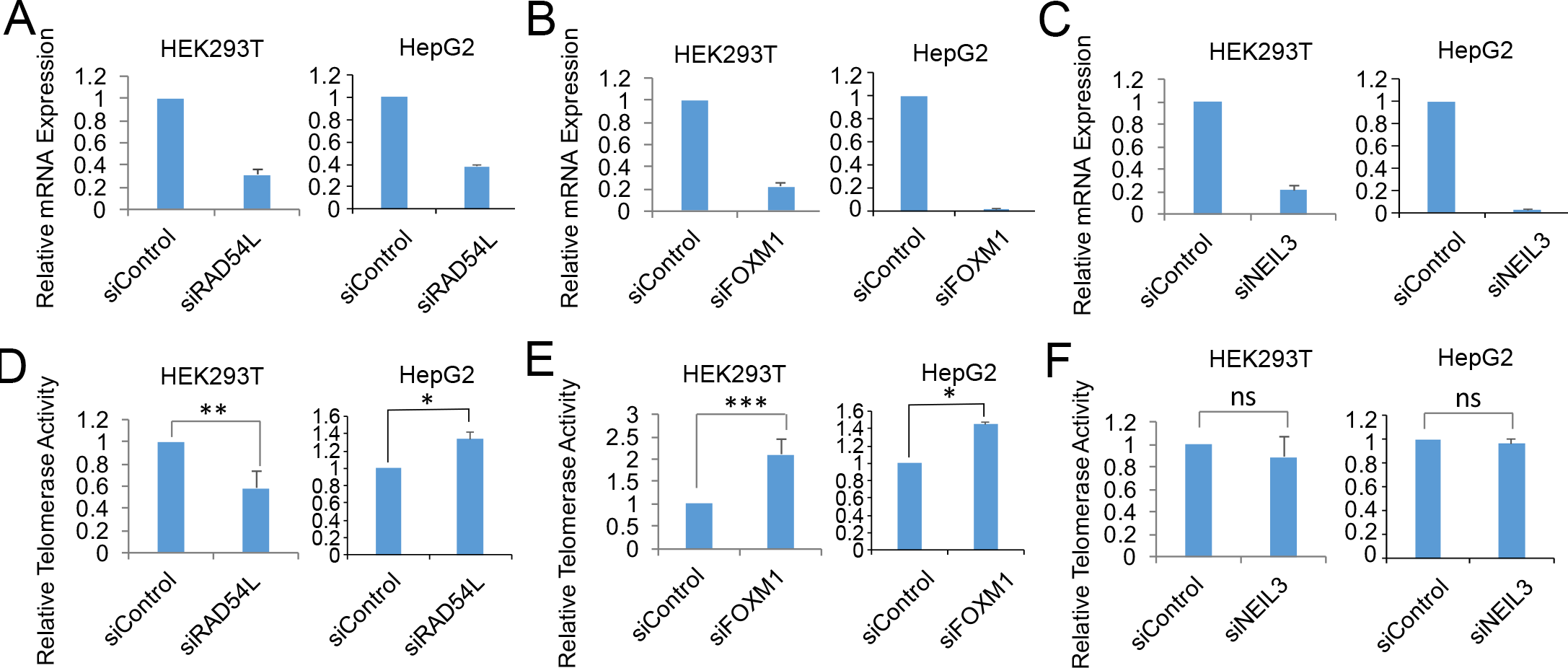

Supplement: Supplementary file 11 — Figure S8. Telomerase activity changes of RAD54L, FOXM1 or NEIL3 knockdown. (TIF 917 kb) [file 12943_2019_1035_MOESM11_ESM.tif]
