# Supplementary material for: Pan-cancer analysis identifies telomerase-associated signatures and cancer subtypes
Source: Mol Cancer. 2019 Jun 10;18:106. doi: 10.1186/s12943-019-1035-x (PMC6556968; doi:10.1186/s12943-019-1035-x)
Supplement: Supplementary file 3 — Table S3. Hub genes of cell cycle/mitotic nuclear division module across eight cancers. (DOCX 102 kb) [file 12943_2019_1035_MOESM3_ESM.docx]

**Supplementary table 3. Hub genes of cell cycle/mitotic nuclear division module across eight cancers.**

| Gene | Hub genes counts* | Mean connectivity value | Mean gene significance | Group |
| --- | --- | --- | --- | --- |
| NCAPG | 8 | 39.18119795 | 3.544108311 | Strong |
| **TPX2** | 8 | 43.10449028 | 3.564129751 | Strong |
| CCNB2 | 7 | 35.3978049 | 2.997283793 | Strong |
| BUB1B | 6 | 35.00794037 | 3.224108511 | Strong |
| CKAP2L | 6 | 34.1318758 | 3.096846617 | Strong |
| DLGAP5 | 6 | 34.80007445 | 3.400942558 | Strong |
| GTSE1 | 6 | 33.44705105 | 3.770517097 | Strong |
| HJURP | 6 | 37.66105223 | 3.229810107 | Strong |
| KIF18B | 6 | 31.54981704 | 4.312257066 | Strong |
| KIF20A | 6 | 31.45268216 | 2.736138619 | Strong |
| NUSAP1 | 6 | 32.53195761 | 3.444965996 | Strong |
| TOP2A | 6 | 35.69695089 | 3.385827252 | Strong |
| ASF1B | 5 | 24.71443856 | 2.016497456 | Strong |
| BUB1 | 5 | 35.63754787 | 3.30258369 | Strong |
| CDCA8 | 5 | 34.29348123 | 3.265049712 | Strong |
| CENPF | 5 | 31.28968964 | 3.295522985 | Strong |
| CEP55 | 5 | 32.95432765 | 2.941543121 | Strong |
| **EXO1** | 5 | 32.50100251 | 2.93159063 | Strong |
| KIF4A | 5 | 36.44936295 | 3.457687163 | Strong |
| KIFC1 | 5 | 34.77063384 | 3.620053772 | Strong |
| NUF2 | 5 | 33.14639945 | 3.464682641 | Strong |
| PLK1 | 5 | 31.19795298 | 3.729637036 | Strong |
| RRM2 | 5 | 31.95242801 | 3.210091721 | Strong |
| SGOL1 | 5 | 33.39902729 | 2.62414501 | Strong |
| SKA1 | 5 | 32.08030845 | 3.369665534 | Strong |
| TTK | 5 | 33.56403201 | 2.623224346 | Strong |
| ASPM | 4 | 30.49716189 | 3.706441217 | Median |
| CDK1 | 4 | 30.17407825 | 2.839449942 | Median |
| CENPA | 4 | 31.73675525 | 3.277872359 | Median |
| **FOXM1** | 4 | 32.2849272 | 3.515016258 | Median |
| KIF11 | 4 | 31.99028509 | 3.596498144 | Median |
| KIF14 | 4 | 31.93995309 | 3.321921816 | Median |
| KIF23 | 4 | 31.22962071 | 3.314392529 | Median |
| MELK | 4 | 31.6181742 | 2.801853169 | Median |
| MKI67 | 4 | 27.8041261 | 3.765614883 | Median |
| NCAPH | 4 | 33.86224798 | 3.347807395 | Median |
| NDC80 | 4 | 27.78620667 | 3.130656225 | Median |
| POLQ | 4 | 28.40210033 | 3.544613063 | Median |
| PRC1 | 4 | 32.1104682 | 2.878819057 | Median |
| ARHGAP11A | 3 | 25.85732919 | 2.473989456 | Median |
| CCNA2 | 3 | 30.88682785 | 3.574017415 | Median |
| CDCA2 | 3 | 23.28223795 | 3.129518921 | Median |
| CENPE | 3 | 26.92099904 | 3.370568058 | Median |
| DEPDC1 | 3 | 30.18187716 | 3.251188119 | Median |
| DTL | 3 | 23.71181266 | 1.835910687 | Median |
| KIF15 | 3 | 27.18066555 | 3.354430018 | Median |
| KIF2C | 3 | 29.62658563 | 2.978117501 | Median |
| MCM10 | 3 | 27.92308963 | 2.634891153 | Median |
| NEK2 | 3 | 30.39381801 | 3.496990945 | Median |
| SKA3 | 3 | 31.06292115 | 3.155948718 | Median |
| TROAP | 3 | 28.42121302 | 4.015379657 | Median |
| UBE2C | 3 | 26.09512468 | 3.548803547 | Median |
| ANLN | 2 | 24.66389263 | 2.491463488 | Median |
| AURKB | 2 | 26.56768859 | 4.140489136 | Median |
| BIRC5 | 2 | 24.36271969 | 2.583450178 | Median |
| CCNB1 | 2 | 23.66420861 | 1.953971999 | Median |
| CDC20 | 2 | 25.29637674 | 2.569195978 | Median |
| CDC25C | 2 | 26.12719747 | 3.169901499 | Median |
| CDC6 | 2 | 23.91627427 | 1.873630431 | Median |
| CDCA3 | 2 | 18.58957048 | 3.319184508 | Median |
| CDCA5 | 2 | 31.07967185 | 3.24894656 | Median |
| ESPL1 | 2 | 23.52890051 | 4.054701083 | Median |
| FAM64A | 2 | 20.93619983 | 2.380907784 | Median |
| FAM72B | 2 | 23.84297013 | 2.848657702 | Median |
| FANCI | 2 | 23.42498299 | 2.318622954 | Median |
| KIAA1524 | 2 | 41.04902535 | 1.745346665 | Median |
| MCM2 | 2 | 15.58791091 | 2.084911902 | Median |
| PLK4 | 2 | 22.38308997 | 3.004214813 | Median |
| RAD51AP1 | 2 | 22.78874924 | 2.192924248 | Median |
| **RAD54L** | 2 | 23.57426423 | 3.159444533 | Median |
| SGOL2 | 2 | 26.65261874 | 1.712577191 | Median |
| SPAG5 | 2 | 16.63050605 | 1.79768646 | Median |
| SPC25 | 2 | 22.15551282 | 3.133177672 | Median |
| STIL | 2 | 27.84930021 | 2.538920822 | Median |
| TICRR | 2 | 18.89670104 | 3.272480951 | Median |
| ZWINT | 2 | 20.48745255 | 2.197253439 | Median |
| AUNIP | 1 | 10.024764 | 1.86142804 | Weak |
| AURKA | 1 | 18.00903599 | 1.947726694 | Weak |
| BRCA1 | 1 | 11.87297983 | 1.424570584 | Weak |
| CASC5 | 1 | 49.63313767 | 1.481313321 | Weak |
| CDC25A | 1 | 13.26368551 | 2.047225601 | Weak |
| CDC45 | 1 | 24.26837924 | 2.647254553 | Weak |
| CDKN3 | 1 | 20.11792853 | 2.552721687 | Weak |
| CENPI | 1 | 17.55952321 | 1.43979585 | Weak |
| CHEK1 | 1 | 11.50584385 | 1.79718843 | Weak |
| CLSPN | 1 | 27.93130603 | 1.777080897 | Weak |
| DEPDC1B | 1 | 19.02587451 | 1.800280254 | Weak |
| DIAPH3 | 1 | 10.52836501 | 1.813184279 | Weak |
| DSCC1 | 1 | 9.651833826 | 1.826509565 | Weak |
| ERCC6L | 1 | 25.86482322 | 2.364882887 | Weak |
| ESCO2 | 1 | 24.13562085 | 2.578435255 | Weak |
| FAM72D | 1 | 20.75114354 | 3.111159589 | Weak |
| FANCD2 | 1 | 16.64460622 | 2.561168191 | Weak |
| FEN1 | 1 | 11.37735804 | 2.449704358 | Weak |
| GINS1 | 1 | 20.96631991 | 1.856919293 | Weak |
| GSG2 | 1 | 24.67427108 | 2.771304905 | Weak |
| KIF18A | 1 | 20.36572001 | 1.675163465 | Weak |
| KPNA2 | 1 | 12.37997801 | 1.426939699 | Weak |
| MAD2L1 | 1 | 18.83046345 | 2.410113775 | Weak |
| MCM6 | 1 | 13.63502341 | 1.478929901 | Weak |
| MTFR2 | 1 | 13.91189666 | 2.750843022 | Weak |
| MYBL2 | 1 | 24.40056627 | 3.121759366 | Weak |
| OIP5 | 1 | 18.93909521 | 2.459852126 | Weak |
| ORC1 | 1 | 20.99337777 | 2.726889875 | Weak |
| PARPBP | 1 | 15.06762741 | 2.319294092 | Weak |
| PBK | 1 | 22.84596927 | 2.717540221 | Weak |
| RACGAP1 | 1 | 21.02908553 | 2.538836904 | Weak |
| RAD51 | 1 | 23.34862841 | 2.507315657 | Weak |
| UHRF1 | 1 | 18.43853912 | 2.352450447 | Weak |

*Hub genes counts: the number of cancers detected corresponding gene was hub gene in cell cycle/mitotic nuclear division module (top 5%).
