# Supplementary material for: Pan-cancer analysis identifies telomerase-associated signatures and cancer subtypes
Source: Mol Cancer. 2019 Jun 10;18:106. doi: 10.1186/s12943-019-1035-x (PMC6556968; doi:10.1186/s12943-019-1035-x)
Supplement: Supplementary file 4 — Table S1. Number of cancer cases analyzed in this study (DOCX 17 kb) [file 12943_2019_1035_MOESM4_ESM.docx]

**Additional file 4: Table S1 Number of cancer cases analyzed in this study.**

| Cancer | Somatic Mutation | | DNA Methylation | | mRNA expression | | miRNA expression | | Somatic CNA | |
| --- | --- | --- | --- | --- | --- | --- | --- | --- | --- | --- |
|  | TERT^high^ | TERT^low^ | TERT^high^ | TERT^low^ | TERT^high^ | TERT^low^ | TERT^high^ | TERT^low^ | TERT^high^ | TERT^low^ |
| BRCA | 582 | 149 | 472 | 113 | 582 | 149 | 480 | 125 | 582 | 149 |
| LUAD | 394 | 58 | 348 | 50 | 394 | 58 | 342 | 49 | 394 | 58 |
| LIHC | 145 | 32 | 145 | 32 | 145 | 32 | 145 | 32 | 145 | 32 |
| KIRC | 129 | 153 | 82 | 114 | 129 | 153 | 63 | 78 | 129 | 153 |
| KIRP | 29 | 69 | 27 | 63 | 29 | 69 | 29 | 69 | 29 | 69 |
| THCA | 73 | 263 | 73 | 263 | 73 | 263 | 73 | 263 | 73 | 263 |
| LGG | 189 | 35 | 189 | 35 | 189 | 35 | 189 | 35 | 189 | 35 |
| SARC | 70 | 106 | 70 | 106 | 70 | 106 | 70 | 106 | 70 | 106 |
