# Supplementary material for: Pan-cancer analysis identifies telomerase-associated signatures and cancer subtypes
Source: Mol Cancer. 2019 Jun 10;18:106. doi: 10.1186/s12943-019-1035-x (PMC6556968; doi:10.1186/s12943-019-1035-x)
Supplement: Supplementary file 9 — Table S2. Enrichment of validated telomere maintenance related Genes within the cell cycle/mitotic nuclear division module genes. (DOCX 14 kb) [file 12943_2019_1035_MOESM9_ESM.docx]

**Additional file 9: Table S2. Enrichment of validated telomere maintenance related Genes within the cell cycle/mitotic nuclear division module genes**

| Cancer type | Cell cycle/mitotic nuclear division | Validated TM related  Genes enriched *P* value |
| --- | --- | --- |
| BRCA | Blue module (N=697) | 6.54975e-05 |
| KIRC | Greenyellow module (N=318) | 0.0007214135 |
| KIRP | Magnenta module (N=537) | 0.001419825 |
| LGG | Yellow module (N=746) | 2.468846e-06 |
| LIHC | Blue module (N=1272) | 0.001474389 |
| LUAD | Turquoise module (N=2034) | 0.06327812 |
| THCA | Skyblue&oranged4 module (N=172) | 6.201093e-07 |
| SARC | Green module (N=718) | 9.071e-05 |

*P*-values represent the hypergeometric probability of the overlap between each cell cycle/mitotic nuclear division module in the network described in this study and the validated TM related genes in the study by Delia M. Braun et al. 2017. In THCA there has two modules have functional involvement with cell cycle/mitotic nuclear division, so we simply merge the genes to generate a new gene set for enrichment analysis.
