# Supplementary material for: Pan-cancer analysis identifies telomerase-associated signatures and cancer subtypes
Source: Mol Cancer. 2019 Jun 10;18:106. doi: 10.1186/s12943-019-1035-x (PMC6556968; doi:10.1186/s12943-019-1035-x)
Supplement: Supplementary file 12 — Table S4. Summary of cox regression results. (DOCX 14 kb) [file 12943_2019_1035_MOESM12_ESM.docx]

Additional file 12: Table S4 Summary of cox regression results

| Predictor | BRCA | | KIRC | | KIRP | | LGG | | LIHC | | LUAD | | SARC | | THCA | |
| --- | --- | --- | --- | --- | --- | --- | --- | --- | --- | --- | --- | --- | --- | --- | --- | --- |
|  | HR  (95% CI) | P value | HR  (95% CI) | P value | HR  (95% CI) | P value | HR  (95% CI) | P value | HR  (95% CI) | P value | HR  (95% CI) | P value | HR  (95% CI) | P value | HR  (95% CI) | P value |
| TERT expression | 1.37 (0.76-2.44) | 0.293 | 1.41  (0.70-2.84) | 0.330 | 3.23  (1.08-9.67) | **0.0359** | 0.87  (0.43 -1.73) | 0.682 | 1.35  (0.68-2.66) | 0.389 | 0.80  (0.49 -1.32) | 0.389 | 2.61  (1.56 -4.38) | **0.000283** | 2.61  (1.56 -4.38) | **0.000108** |
| Telomerase activity predictor | 1.62 (0.98-2.69) | 0.062 | 0.64  (0.32-1.27) | 0.062 | 0.40  (0.13-1.23) | 0.11 | 8.59  (4.75-15.53) | **1.08E-12** | 0.93  (0.57-1.51) | 0.762 | 1.62  (1.14-2.30) | **0.00715** | 1.13  (0.65-1.97) | 0.662 | 0.51  (0.13-1.87) | 0.308 |
| Random forest predictor | 1.79 (1.06-3.02) | **0.029** | 3.72 (1.74-7.94) | **6.77 E-04** | 12.19  (2.69-55.13) | **0.00117** | 5.50  (2.94-10.29) | **1.02E-07** | 2.69 (1.56-4.66) | **4.08E-04** | 1.60  (1.11-2.32) | **0.0119** | 1.65  (0.93-2.94) | 0.087 | 2.40  (0.74-7.81) | 0.145 |

HR, hazard ratio; CI, confident interval; P value < 0.05 are bold.
